# Alterations in Metabolome and Microbiome Associated with an Early Stress Stage in Male Wistar Rats: A Multi-Omics Approach

**DOI:** 10.3390/ijms222312931

**Published:** 2021-11-29

**Authors:** Julia Hernandez-Baixauli, Pere Puigbò, Nerea Abasolo, Hector Palacios-Jordan, Elisabet Foguet-Romero, David Suñol, Mar Galofré, Antoni Caimari, Laura Baselga-Escudero, Josep M. Del Bas, Miquel Mulero

**Affiliations:** 1 Eurecat, Centre Tecnològic de Catalunya, Unitat de Nutrició i Salut, 43204 Reus, Spain; julia.hernandez@eurecat.org (J.H.-B.); pere.puigbo@eurecat.org (P.P.); antoni.caimari@eurecat.org (A.C.); laura.baselga@eurecat.org (L.B.-E.); 2 Department of Biochemistry and Biotechnology, Universitat Rovira i Virgili, 43007 Tarragona, Spain; 3 Department of Biology, University of Turku, 20014 Turku, Finland; 4 Eurecat, Centre Tecnològic de Catalunya, Centre for Omic Sciences (COS), Joint Unit Universitat Rovira i Virgili-EURECAT, 43204 Reus, Spain; nerea.abasolo@eurecat.org (N.A.); hector.palacios@eurecat.org (H.P.-J.); elisabet.foguet@eurecat.org (E.F.-R.); 5 Eurecat, Centre Tecnològic de Catalunya, Digital Health, 08005 Barcelona, Spain; david.sunol@eurecat.org (D.S.); mar.galofre@eurecat.org (M.G.); 6 Nutrigenomics Research Group, Department of Biochemistry and Biotechnology, Universitat Rovira i Virgili, 43007 Tarragona, Spain

**Keywords:** early stress, biomarker, animal model, chronic unpredictable mild stress, metabolome, microbiome, energy disruption

## Abstract

Stress disorders have dramatically increased in recent decades becoming the most prevalent psychiatric disorder in the United States and Europe. However, the diagnosis of stress disorders is currently based on symptom checklist and psychological questionnaires, thus making the identification of candidate biomarkers necessary to gain better insights into this pathology and its related metabolic alterations. Regarding the identification of potential biomarkers, omic profiling and metabolic footprint arise as promising approaches to recognize early biochemical changes in such disease and provide opportunities for the development of integrative candidate biomarkers. Here, we studied plasma and urine metabolites together with metagenomics in a 3 days Chronic Unpredictable Mild Stress (3d CUMS) animal approach that aims to focus on the early stress period of a well-established depression model. The multi-omics integration showed a profile composed by a signature of eight plasma metabolites, six urine metabolites and five microbes. Specifically, threonic acid, malic acid, alpha-ketoglutarate, succinic acid and cholesterol were proposed as key metabolites that could serve as key potential biomarkers in plasma metabolome of early stages of stress. Such findings targeted the threonic acid metabolism and the tricarboxylic acid (TCA) cycle as important pathways in early stress. Additionally, an increase in opportunistic microbes as virus of the *Herpesvirales* was observed in the microbiota as an effect of the primary stress stages. Our results provide an experimental biochemical characterization of the early stage of CUMS accompanied by a subsequent omic profiling and a metabolic footprinting that provide potential candidate biomarkers.

## 1. Introduction

Psychological stress disorders have dramatically increased in recent decades, becoming a prevalent global health problem. Nowadays, it affects the lives of almost 300 million people worldwide suffering from a range of different stress disorders [1]. In line with this, the World Health Organization (WHO) estimates that stress disorders, anxiety and depression cost to the global economy around USD 1 trillion each year due to lost productivity [1]. Generally, stressful events are thought to influence the pathogenesis of other non-communicable diseases (NCDs) by causing negative affective states (e.g., feelings of anxiety and depression) [2]. During stressful events, two endocrine response systems are activated: the hypothalamic–pituitary–adrenocortical axis (HPA) and the sympathetic–adrenal–medullary (SAM) system. Thus, prolonged or repeated activation of the HPA and SAM systems can interfere with a broad range of physiological processes, resulting in an increased risk of NCDs, particularly cardiovascular diseases, in addition to the traditional psychiatric disorders related with stressful events [3].

The diagnosis of stress disorders, like all psychiatric disorders, is mainly based on symptom checklists and psychological questionnaires referring to a single diagnosis, while patients commonly present symptoms that fit multiple diagnoses [4]. Therefore, the identification of biomarkers and altered metabolic pathways in stress disorders are necessary to gain better insights into the mechanisms that promote metabolic alterations that usually come along with stressful events. This knowledge will allow either: (a) early and accurate diagnosis (by means of biomarkers discovery) and/or, (b) prevention treatments, tailored interventions and general treatments based on the direct or indirect restoration of metabolic parameters.

Regarding the novel approaches for the identification of new potential biomarkers, omics profiling seems to be a promising methodology for the identification of early biochemical changes in disease and thus provides an opportunity to discriminate a footprint of candidate biomarkers that can favor the initiation of earlier interventions; for example, through personalized nutrition and life-style modifications to avoid future drug treatments [5,6]. In this sense, the most relevant biological material for the study of biomarkers in psychiatric disorders derives from the brain [7]. Nevertheless, human brain samples are only available for post-mortem analysis; in consequence, animal models are essential for translating these results to more feasible tissues for the detection of molecular pathways implicated in the pathology and for finding candidate biomarkers of stress disorders [8]. Thus, the use of plasma, serum and urine has been increasing in the metabolomic study of mental disorders, which also provides valuable information about the effect of the disorder throughout the body, as the brain is engaged in all physiological functions of the body [9].

Metabolomic approaches point out that oxidative stress, alterations in lipid and energy metabolism (i.e., mitochondrial regulation), glutamine metabolism and neurotransmitters metabolism could be involved in stress disorders [10]. These metabolic alterations could overlap with depressive disorder—which often occur simultaneously in individuals with stressful events—where changes in the glutamate–glutamine cycle, as well as changes in lipid and energy metabolism, have been found to be related to the pathogenesis of major depressive disorder [11]. For this reason, it could be interesting to study the early stress episodes to further differentiate between depression and psychological stress. The Chronic Unpredictable Mild Stress (CUMS) model is an experimental approach commonly used to simulate the core behavioural characteristics of human depression for investigating the pathophysiology and assisting in diagnosis [12]. Therefore, the study of CUMS during an initial short period could provide a valuable tool for studying the effects of early stressful events before the animals develop depression disorder. Previous metabolomic studies have fully profiled the metabolic patterns of the rodent CUMS model [13]; nevertheless, it is of interest to distinguish between a classical CUMS and an early CUMS to determine early biomarkers of nerve-wracking events.

To summarize, depressive-related states are the most prevalent psychiatric disorder, but no early metabolic biomarkers have been clearly identified for their early diagnosis, accurate patient subcategorization, treatment or effective prevention. In this study, we explore and interrogate a CUMS experimental model during a period of 3 days (3d) to unravel the neurobiological underpinnings and to identify candidate biomarkers and affected pathways of harsh stress episodes, using the metabolome of different biofluids (plasma and urine) and the microbiome.

## 2. Results

### 2.1. Characterization of the Early Stress Stage in Male Wistar Rats

**Figure 1:**
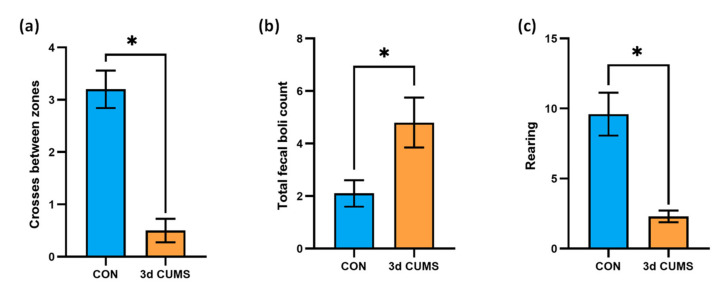
Evaluation of the anxiety-like behaviour on the OFT test in the early stress stage. CON and 3d CUMS (*n* = 10 animals per group) rats were subjected to the OFT for 5 min. (**a**) Number of crosses between inner and outer zone. (**b**) Total number of fecal boli deposits. The number of defecations was counted by the researcher after the rats were removed. (**c**) Rearing behaviour. The statistical comparisons among groups were conducted using Student’s t test; the statistically significant *p*-values versus CON (*p* < 0.05) are highlighted with an asterisk (*).

Thigmotaxis is “wall-hugging” behaviour. For example, this conduct is frequently exhibited by humans when they enter into an elevator with strangers. In an open field experiment, rodents will typically exhibit less thigmotaxis as they become acclimatized to the chamber. In this regard, the anxiety-like behaviour level of the animals was measured on the open field test (OFT) and is summarised in Figure 1. The number of entries and the total time in the zones were determined to analyse such behaviour (anxious or fearful animals will spend less time in the centre of the field and more time next to the walls, yielding a decreased centre-to-total time ratio). Interestingly, the number of crosses between zones decreased in the 3d CUMS group (*p*-value = 0.02), indicating less motor activity than in the control (CON) group (Figure 1a). Generally, the total time in the outer zone was lower than the total time spent in the inner zone in all the animals. However, the total time in the inner zone was not statistically different (data not shown). Additionally, to further evidence an anxiety-like behaviour, we observed that the 3d CUMS group exhibited a significant increase in fecal boli deposits (*p*-value = 0.02, Figure 1b), and the rearing pattern was significantly decreased in 3d CUMS group (*p*-value < 0.01, Figure 1c); such rearing evaluation consisted in the assessment of the total number of erect postures, thorough the full test period, adopted by the rodent with the intention of exploring.

The main endocrine hormones related to the stress response were measured in plasma (i.e., corticosterone and serotonin) (Table 1). The results showed that both hormones were significantly increased in the 3d CUMS group. In this regard, corticosterone (*p*-value < 0.01) and serotonin (*p*-value = 0.01) were increased in the 3d CUMS group approximately more than six times and four times, respectively.

To obtain a broader description of the rodent, the biometric and plasma parameters were also measured (Table 1). The body weight was constant during the three days of the experiment, and no differences were observed in food intake. Furthermore, no differences were observed in the weight of the analysed tissues. Focusing on the plasma parameters, the 3d CUMS group presented an increase in glucose concentration (*p*-value < 0.01), as well as a rise in triglycerides (TG, *p*-value = 0.2), total cholesterol (TC, *p*-value = 0.06) and non-esterified fatty acids (NEFAs, *p*-value = 0.02).

### 2.2. Plasma Metabolic Profiling and Biomarker Identification

The plasma metabolomic approach was based on a global multiplatform analysis including 138 metabolites (Table S1). This platform was associated with the following biochemical processes: the lipid metabolism (represented as a wide diversity of different triacyclglycerols (TGs), diacylglycerols (DGs), phosphatidylcholines (PCs), cholesterol esters (ChoEs), lysophospholipids (LPCs) and sphingomyelins (SMs), among others); the carbohydrate metabolism (where the main metabolites of tricarboxylic acid cycle (TCA cycle) were included); and the amino acid metabolism. The summaries of the univariate and multivariate analyses are shown in the Table S1. After the Mann–Whitney (MW) test, 23 out 138 metabolites were significantly different, and the subsequent Benjamin–Hochberg (BH) correction highlighted 6 out of 23 different metabolites that were as follows: succinic acid, malic acid, threonic acid, alpha-ketoglutarate, pyruvic acid, cholesterol, oleic acid and 3-hydroxybutitic acid (Table 2).

**Figure 2:**
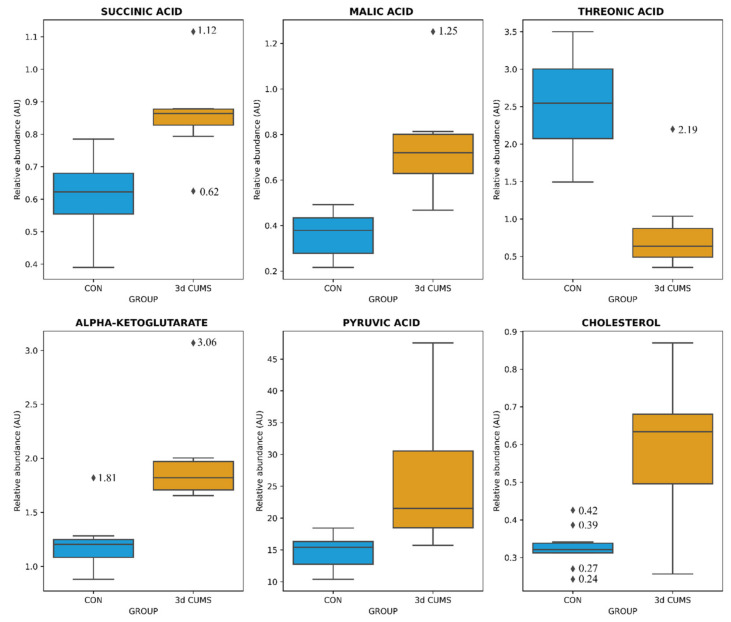
Box-whisker plots of the differential metabolites in the early stress stage. Relative abundance of metabolites (AU) is represented: blue represents CON group and orange the 3d CUMS group (*n* = 10 animals per group). Box denotes 25th and 75th percentiles; line within box denotes 50th percentile (median); whisker denotes standard deviation.

The distribution of the six metabolites were visualized with boxplots to evaluate the distribution between groups and the impact in the early stress stage (Figure 2): Threonic acid was decreased more than three times in the 3d CUMS group, while the other metabolites were increased.

**Figure 3:**
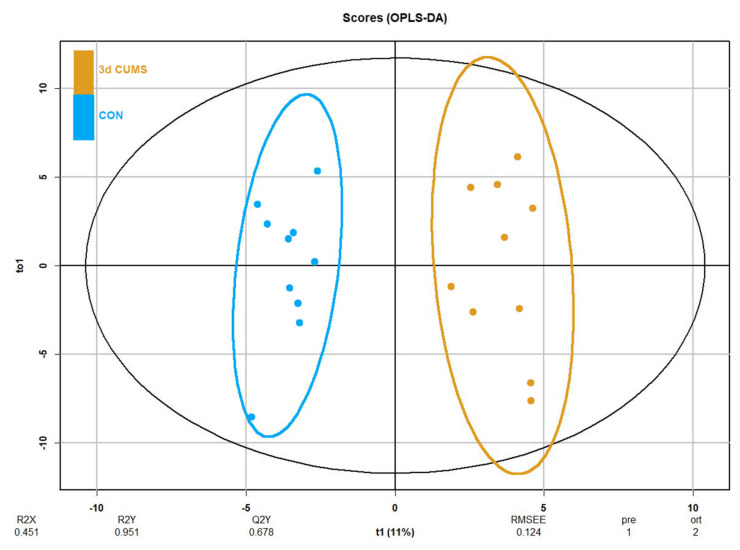
OPLS-DA of plasma metabolomics in the early stress stage. Blue represents the CON group and orange the 3d CUMS group (*n* = 10 animals per group). The Score plot is represented, and it includes the number of components, the cumulative R2X, R2Y and Q2Y.

Despite no clustering being distinguished in the principal component analysis (PCA, Figure S1), differences in orthogonal the partial least squares discriminant analysis (OPLS-DA) were observed between groups (Figure 3). OPLS-DA was performed in parallel to the statistical analysis to assess the prediction power of the key plasma metabolites. The proportion of variance explained by the model (R2X) was 45.1% in the plasma data. The percentage of Y variability explained by the model (R2Y) was 95.1% and the estimation of the predictive performance of the models (Q2) was 67.8%; as it is greater than 50%, the model is considered to have good predictability. The highest variable importance in projection (VIP) values are shown in Table 2, threonic acid (2.6) being the most important metabolite in the model, followed by malic acid, cholesterol, alpha-ketoglutarate and succinic acid. Finally, the feature importance was also assessed using a random forest classifier (RF) to complete the evaluation of the prediction power (Table S1). In this case, the most important feature was the malic acid followed by threonic acid, oleic acid and alpha-ketoglutarate, with values above 0.1.

### 2.3. Urine Metabolic Profiling and Biomarker Identification

**Figure 4:**
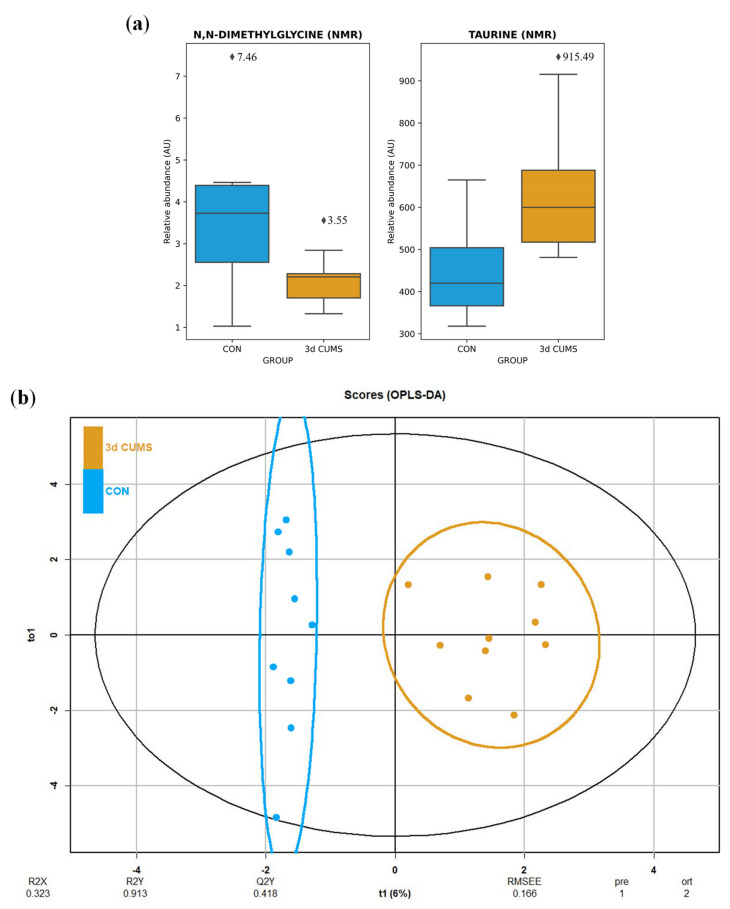
Box-whisker plots and OPLS-DA representation of urine metabolomics in the early stress stage. Blue represents the CON group and orange the 3d CUMS group (*n* = 10 animals per group). (**a**) Box-whisker plots of the major impact metabolites in urine represented by the relative abundance of metabolites (AU). Box denotes 25th and 75th percentiles; line within box denotes 50th percentile; whisker denotes standard deviation. (**b**) OPLS-DA of plasma metabolomics. The score plot that is represented includes the number of components, and the cumulative R2X, R2Y and Q2Y (indicated below the plot).

The urine metabolomic approach, which was based on the untargeted ^1^H-NMR methodology, detected 42 metabolites mainly belonging to the amino acid metabolism (e.g., phenylalanine, tyrosine and tryptophan metabolism; glycine, serine and threonine metabolism; alanine, aspartate and glutamate metabolism; glutathione metabolism; and taurine and hypotaurine metabolism) and the energetic metabolism (e.g., TCA cycle, pyruvate metabolism and glycolysis/gluconeogenesis) (Table S2). The summary of the univariate and multivariate analysis is shown in the Table S2. After the MW test, N,N-dimethylglycine and taurine were significantly altered in the 3d CUMS group versus the CON group. After the BH correction, none of these metabolites remained significantly modified. The distribution was also analysed, noticing a decrease in N,N-dimethylglycine and an increase in taurine (Figure 4a).

Differences were observed between groups in OPLS-DA (Figure 4b), despite no clustering being distinguished in the PCA (Figure S2). The proportion of variance explained by the OPLS-DA model (R2X) was 32.3% in the urine data. The percentage of Y variability (R2Y) was 91.3% and the estimation of the predictive performance (Q2) was 41.8%. In this case, the predictive power in urine metabolites is not strong enough to discriminate between groups. The highest VIP values are shown in Table S2, being the before-mentioned metabolites the most important in urine with higher VIP values. In this case, the most important features were also N,N-dimethylglycine (0.21) and taurine (0.18) applying RF to elucidate the evaluation of this metabolites (Table S2).

### 2.4. Microbiome Profiling

**Figure 5:**
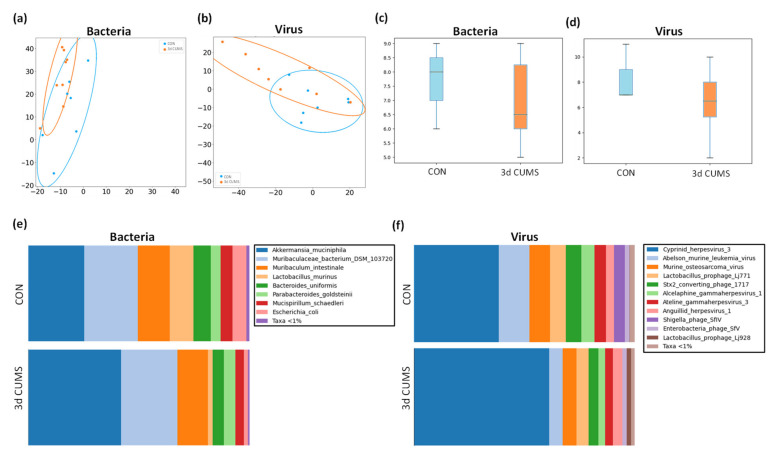
Summary of the microbiome statistical analysis in the early stress stage. Blue represents the CON group and orange the 3d CUMS group (*n* = 8 animals per group). Beta diversity: PCA plot calculated by Aitchison distance for bacteria (**a**) and viruses (**b**). Alpha diversity (AU): chao1 index in bacteria (**c**) and viruses (**d**). Taxonomic differences represented as relative distribution of species in bacteria (**e**) and viruses (**f**); these figures show a bar graph at the level of both bacterial and viral species (relative %) comparing the animals in all groups.

The taxonomic assignment detected the presence of the most abundant microbes in the cecum section to evaluate the highest variability and diversity of the gut tract (i.e., bacteria, viruses and <1% of other microbes). Making a general overview related to the abundance of microbes, we can see that 78% of the generated readings were assigned to bacteria and 22% to viruses in the CON group, and in the case of the 3d CUMS group, the readings assigned to bacteria were slightly decreased to 69% and the viruses were increased to 31% in comparison to CON (not statistically significant). The beta diversity, which is represented by a PCA of Aitchison distances, were highly overlapped between bacterial groups (Figure 5a) and between viruses (Figure 5b). In this regard, the PERMANOVA showed a tendency in bacteria (F = 1.89, *p*-value = 0.09) indicating differences in bacteria composition/beta diversity, while there were no significant differences in viruses (F = 0.93, *p*-value = 0.5). The alpha diversity (measure of richness in the same group) showed a tendency to decrease in the microbiome of the 3d CUMS group, without being statistically significant in neither bacteria (Figure 5c) nor viruses (Figure 5d).

Regarding the bacterial microbiome, the communities of both groups were mostly formed by the phyla *Bacteroidetes* (CON: 51% and 3d CUMS: 49%), *Verrucomicrobia* (CON: 26% and 3d CUMS: 42%), *Firmicutes* (CON: 12% and 3d CUMS: 4%), *Proteobacteria* (CON: 6% and 3d CUMS: 2%), and *Deferribacteres* (CON: 5% and 3d CUMS: 4%). Although these differences were not statistically significant, interesting results could be found on the magnitude of the different phyla. Thus, the relative abundance of *Verrumicrobia* was increased almost twice, while *Firmicutes* and *Proteobacteria* decreased three times in the 3d CUMS group. Focusing on bacterial species (Figure 5e), 12 species were found to have a relative abundance above 0.01% (Table S3). Interestingly, the *Akkermansia muciniphila* species was the most abundant one (CON: 26% and 3d CUMS: 42%), being the main species implicated in the increase in the *Verrucomicrobia* phylum in both groups (Figure 5a). However, the difference was not significant, even though the values almost doubled (*p*-value = 0.1 and *q*-value = 0.4). The higher increase in *Akkermansia muciniphila* indirectly affects the relative abundance of other species, for example, *Lactobacillus murinus* (CON: 11% and 3d CUMS: 2%), *Escherichia coli* (CON: 6% and 3d CUMS: 2%) or *Bacteroides uniformis* (CON: 8% and 3d CUMS: 5%) (Figure 5a).

In the case of the virus microbiome, the communities of both groups were mostly formed by the following orders: *Herpesvirales* (CON: 54% and 3d CUMS: 73%), *Ortervirales* (CON: 24% and 3d CUMS: 12%) and *Caudovirales* (CON: 23% and 3d CUMS: 14%). Thus, the relative abundance of *Herpesvirales* increased almost 1.5 times in the 3d CUMS group; however, *Ortervirales* and *Caudovirales* decreased twice in the same group. Focusing on virus species (Figure 5f), 13 species were found to have a relative abundance above 0.01% (Table S4). In both groups, the most represented virus was an uncharacterized *herpesvirus* (CON: 39% and 3d CUMS: 61%) that was increased in the 3d CUMS group (*p* = 0.02). This virus is the main species implicated in the *Herpesvirales* order (Figure 5b). Other relative abundant species were *Abelson murine leukemia* virus (CON: 14% and 3d CUMS: 6%), *Murine osteosarcoma* virus (CON: 9% and 3d CUMS: 6%), *Lactobacillus prophage Lj771* (CON: 7% and 3d CUMS: 5%), *Stx2 converting phage 1717* (CON: 7% and 3d CUMS: 4%), *Alcelaphine gammaherpesvirus* (CON: 6% and 3d CUMS: 3%) and *Ateline gammaherpesvirus* (CON: 5% and 3d CUMS: 4%), among others (Figure 5b).

### 2.5. Multi-Omics Data Integration

**Figure 6:**
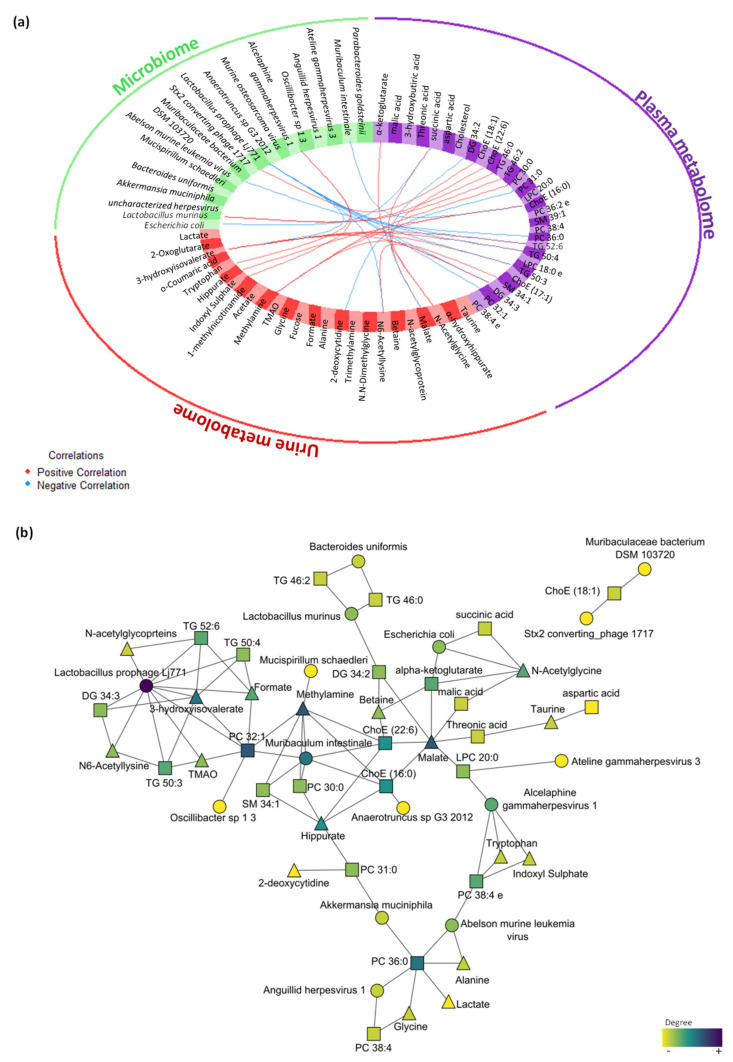
Multi-omics integration of plasma metabolome, urine metabolome and microbiome in the early stress stage. (**a**) Circos plot output from DIABLO. Each quadrant indicates the type of features: plasma metabolites (purple), urine metabolites (red), bacteria species (green) and virus species (orange); lines indicate measure of association (correlation), either positive or negative. (**b**) Further visualization of the network from DIABLO using Cytoscape. The shape of the features indicates the type of feature: plasma metabolites (square), urine metabolites (triangle) and metagenomics (circle). The colour indicates the degree of each feature in the network (i.e., nodes with more connections). Abbreviations: DG, diacylglycerol; ChoE, cholesterol ester; TG, triglyceride; PC, phosphatidylcholine; SM, sphingomyelin; LPC, lysophospholipid.

The multi-omics integrative analysis with Data Integration Analysis for Biomarker discovery using Latent cOmponents (DIABLO) identified a highly associated profile of eight plasma metabolites (threonic acid, alpha-ketoglutarate, malic acid, 3-hydroxybutiric acid, DG 34:2, succinic acid, aspartic acid and cholesterol); six urine metabolites (taurine, α-hydroxyhippurate, N-acetylglycine, malic acid, N,N-dimethylglycine and betaine); and five microbes, including bacteria and viruses (*Escherichia coli*, *Lactobacillus murinus*, *Akkermansia muciniphila*, uncharacterized *Herpesvirus* and *Bacteroides uniformis*). This analysis revealed a high correlation between data sets with coefficients above 0.6 (Figure S3a), specifically between the plasma metabolome that correlated with the urine metabolome (r = 0.82) and microbiome (r = 0.81). Moreover, the data sets were able to discriminate between groups (Figure S3b), highlighting the relationship between plasma and the urine metabolome (Figure S3c). The variable effect in the first component and the impact of each feature in the data set are shown in Figure S4a,c,e for plasma metabolomics, urine metabolomics and metagenomics, respectively. The correlation between the variables from the three different blocks is shown in Figure 6a (cut-off was set at 0.7). Further visualization in Cytoscape revealed the highest correlations between the different omics (Figure 6b).

To evaluate the performance of the proposed omics profile, the overall error was calculated as 0.2 in the first component. Additionally, the receiver operating characteristic (ROC) curve analysis showed that the optimal omics profile with the combination of eight plasma metabolites effectively separated both groups with an area under the ROC curve (AUC) of 1 (*p*-value < 0.01, Figure S4b). A combination of six plasma metabolites optimally dichotomized the groups with an AUC of 0.86 (*p*-value = 0.02, Figure S4d). In the metagenomics data, the combination of five bacteria and viruses grouped the animals with an AUC of 0.92 (*p*-value < 0.01, Figure S4f). These results support the above-selected features as a representative omics profile of the early stress stage.

## 3. Discussion

In the present study, differences in the patterns of anxiety-like behaviour were found in 5–6 months old male Wistar rats in the early stress stage. Generally, younger animals (with ages between 6 and 9 weeks) have been used for these kinds of experiments. These animals could be more resilient and adaptive to stressful events than the older ones [14,15]. Interestingly, even rat and mice pups have also been used to study anxiety-like behaviour [16]. However, it is not until 5–6 months old that rats are fully grown in terms of social competence, brain development and musculoskeletal maturity [17]. Therefore, the use of adult animals is especially interesting because a lot of physiological and molecular phenomena are still changing over the first 3 months of age [18]. Thus, increasing data indicate that important changes in the emotional behaviour occur with aging [19]. The evaluation of exploratory behaviour and general activity was evaluated showing an alteration in anxiety-like behaviour that was characterized by a decrease in motor activity (decrease in the number of crosses between zones in the OFT) in the 3d CUMS group [20]. Furthermore, defecation was increased and was inversely correlated with rearing, suggesting adverse conditions as an initial depressive behaviour [21]. The increased levels of corticosterone and serotonin confirmed the activation of the HPA axis and thus a high increase in the activity of endocrine pathways that could lead to NCDs [3]. In this regard, it has been found in different studies that corticosterone has an impact on hepatic lipid metabolism, energy metabolism and the subsequent metabolite profile [22,23]. Moreover, some biochemical parameters were increased (i.e., glucose concentration) in the 3d CUMS group, as other evidence of the activation of the endocrine response from a metabolic point of view.

**Figure 7:**
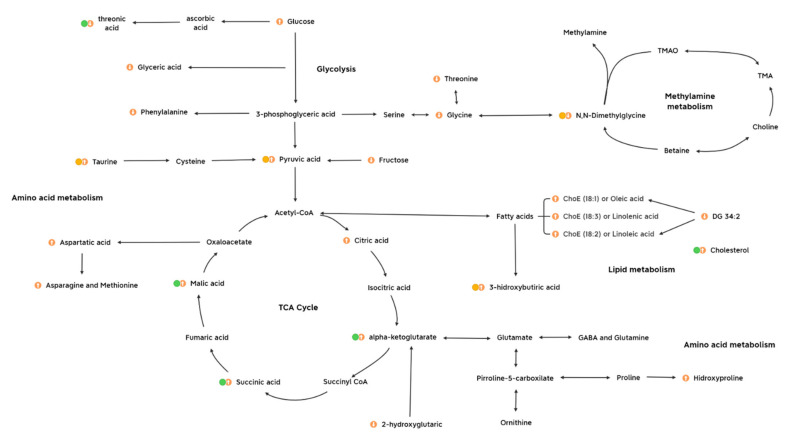
Metabolic profiling of candidate biomarkers and the main metabolic pathways implicated in the early stress stage. It is presented the metabolites with highest influence on the model (match in all the statistical methods, green dot), influence on metabolites (partial match in the statistical methods, yellow dot) and other metabolites presenting an impact on metabolism. The up- and downregulated metabolites are indicated with up and down arrows, respectively. Abbreviations: DG, diacylglycerol; ChoE, cholesterol ester; TMAO, trimethylamine N-oxide; TMA, trimethylamine; GABA, gamma aminobutyric acid.

The metabolome was investigated for the elucidation of neurobiological underpinnings and for the identification of candidate biomarkers in the affected pathways in this early stress stage approach (summarized in Figure 7). Interestingly, one main impact on the metabolome was characterized by a decrease in threonic acid, which is the major breakdown product of ascorbate, being a distinctive metabolite of the 3d CUMS approach compared to other metabolomic classic CUMS studies [13]. In this sense, the presence of high levels of ascorbate in neurons seems to be related to high levels of aerobic respiration rates that could lead to superoxide production and prooxidant effects in mitochondria [24]. Additionally, the control of threonic acid levels have been described as a promising strategy for predicting the subtypes of depressive disorders [25]. In line with this, different strategies have been tailored, focusing on threonic acid, to try to improve multiple brain disorders (e.g., dietary treatment with magnesium-L-threonate) [26].

Several studies have demonstrated a potential link of stressful events with the alteration of energy metabolism thought the stress response [27]. In the 3d CUMS group, increased key intermediate products of the TCA cycle are indicative of the overstimulation of this cycle (i.e., alpha-ketoglutarate, malic acid and succinic acid) due to the stress response. This fact differentiates the 3d CUMS from the classical CUMS that is associated with energy disruption or deficiency (being one of the most represented depressive symptoms associated to the reduction of the activity and curiosity in animal models) [26]. These results supports the idea that, lately, mitochondrial organelles and the energetic metabolism are emerging as modulators of anxiety-related behaviour both in rodent and human studies [28]. One of the intermediate products of the TCA cycle, alpha-ketoglutarate, is an important source of neurotransmitters (i.e., glutamate and gamma aminobutyric acid (GABA)), which are the major brain neurotransmitters mediating excitatory and inhibitory signalling, respectively [29]. Moreover, the alpha-ketoglutarate pathway can synthesize other important amino acids, including proline, hydroxyproline and ornithine, which tend to be increased in the 3d CUMS group as in the case of CUMS model [13]. Related to the energy metabolism, pyruvic acid, which is increased in the 3 CUMS group, is the end-product of glycolysis, a major substrate for oxidative metabolism and a branching point for glucose, lactate, fatty acid and amino acid synthesis [30]. In previous CUMS studies, pyruvic acid was disturbed without being significant, and for this reason, we suggest that the increased pyruvic acid is related to the early response to stress instead of depression when pyruvic acid returns to its normal levels, leading to other metabolic changes [31].

The slightly plasmatic increase in 3-hydroxybutiric acid, a known ketone body, reflects an increase in energy production through fatty acid oxidation, supporting the idea that the disruption of carbohydrate and energy metabolism might be disrupted in the 3d CUMS group. Hereof, the production of ketone bodies has been shown to have a positive influence on the production of GABA, which is illustrated by reduced plasma levels in depressed patients [25]. In contrast, in the present study, we suggest that, paradoxically to depression, an increment in ketone bodies could be associated with stress states. Furthermore, the increased urine excretion of taurine has been highly related to the stress response [32]. This amino acid plays a vital role in the central nervous system acting as a neurotransmitter and in the regulation of oxidative and energy metabolism. In this regard, taurine raises fatty acid oxidation and ketone bodies levels, which is connected with the increase in fatty acids and other related lipids and the increase in 3-hydroxybutiric acid, as has been previously discussed [33]. According to previous studies, taurine embraces antidepressant and anxiolytic activities in different animal models using different doses and types of administration [34].

In studies focused on lipidomics, the connection between lipids, neuronal signaling, and NCDs stands out [35]. In our case, we observed an increase in this group of metabolites characterized by the cholesterol increase, as well as others lipids less relevant in the experimental statistical approach, such as the case of complex lipids (i.e., DG 34:2) and polyunsaturated fatty acids (PUFAs) (i.e., ChoE (18:3), also known as linolenic acid, and ChoE (18:2), also known as linoleic acid) and monounsaturated fatty acid (MUFA) (i.e., ChoE (18:1), also known as oleic acid). Those fatty acids are precursors of other important fatty acids, supporting the evidence of a potential crucial role of membrane lipids and lipid oxidation in mental disorders [24]. In fact, disturbances in those PUFAs, precursors of arachidonic acid and docosahexaenoic acid, were associated with inflammation response and further complications as type 2 diabetes and cardiovascular diseases that were also altered in CUMS studies [31].

Interestingly, a major finding regarding the metabolites excreted in urine is their relation to methylamine metabolism that has been critically related to stressed phenotype through the decrease in choline (precursor of neurotransmitters in the brain) [36]. Furthermore, methylamines have been defined as microbiota-derived metabolites [37]. N,N-dimethylglycine and related metabolites of methylamine metabolism are mainly produced when choline is catabolized into other metabolites via gut microbiota [38], and they are finally excreted in urine. This fact could suggest that the proposed early stress stage may be associated with alterations on the intestinal microorganism populations.

Accordingly, the gut microbiota has been widely studied in psychological disorders, and it has been proposed as a pivotal axis in the regulation of anxiety-like behaviour. Even though the 3d CUMS group was absent of important differences in the gut microbiome, there were some microbes and metabolites that were altered related to a hypothetical gut microbiome disruption. Recent studies in rats subjected to CUMS revealed that the changes in the gut microbiome were associated with the dysregulation of plasma metabolites related to the metabolism of glycerophospholipids, glycerolipids, fatty acyls and sterols [39]. In consequence, this metabolite alteration could be caused by the synergistic effect of the altered microbiome and the stress response. Blacher and colleagues identified that nicotinamide (a vitamin enrolled in the production of steroid hormones synthesized by the adrenal gland and stress-related hormones) is produced by *Akkermansia muciniphila*, and when it was injected into diseased mice, it improved their anxiety-like behaviour [40]. This vitamin may be involved in minimizing oxidative stress and the consequent preservation of neuronal health. Accumulating evidence suggests that gut microbes can also produce metabolites with high neuroactive potential (neurotransmitters) [41]. The interaction between gut microbiota and gut hormones has been greatly evidenced in gut–brain cross-talk [42]. Recently, *Bacteroides* spp. have been confirmed as producers of GABA as a mechanism of stress tolerance in humans [43]. Furthermore, other bacteria taxa, which were altered in our experimental approach, such as *Escherichia coli* and lactic acid bacteria have been found to also produce neurotransmitters such as serotonin [44] and GABA [45], respectively. Interestingly, it has been described an increment in opportunistic microbes [46] as in the case of some bacteria (e.g., *Lactobacillus*) and some viruses (e.g., *Herpesvirus* order) as consequence of stressful events.

Finally, previous studies that profiled stress for finding potential candidate biomarkers presented some drawbacks when trying to obtain pure stress biomarkers because of the existing similarities between early stress stages and the development of depressive disorder [8,11,47,48,49,50,51]. In our study, a comparison between univariate and multivariate analyses, RF and multi-omics integration was performed to check the robustness among methods of the candidate biomarkers and try to determine the essence of stress (Table S5). The full matching metabolites are malic acid, threonic acid, alpha-ketoglutarate, succinic acid and cholesterol in plasma metabolomics (Figure 7), showing their importance as key metabolites in the 3d CUMS study. Additionally, pyruvic acid and 3-hydroxibutiric acid in plasma metabolomics and N,N-dimethylglycine and taurine in urine metabolomics match as candidate biomarkers in two different statistical methodologies (Figure 7). Globally, considering the results of the present 3d CUMS study and previous CUMS studies, we have observed that energy metabolism has a greater impact together with fatty acids, while in CUMS, energy metabolism does not have much impact while there is greater alteration of amino acids associated with the monitoring of depression [31,52].

## 4. Materials and Methods

### 4.1. Animal Experimental Design

A total of 20 22-week-old male Wistar rats (Harlan Laboratories, Barcelona, Spain) were housed individually with a shelter (i.e., cardboard tube) to enrich the cage environment, under fully controlled conditions including temperature (22 ± 2 °C), humidity (55 ± 5%) and light (12 h light–dark cycle and lights on at 9:00 am). All rats were given standard chow diet and tap water ad libitum. The Animal Ethics Committee of the University Rovira i Virgili (Tarragona, Spain) approved all the procedures (code 10049). The experimental protocol followed the “Principles of Laboratory Care” and was carried out in accordance with the European Communities Council Directive (86/609/EEC).

**Figure 8:**
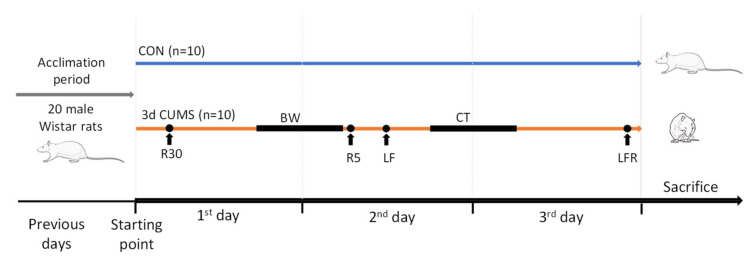
Schematic representation of an early stress stage based on a 3 days CUMS showing the CON and 3d CUMS groups (*n* = 10 animals per group) during the 3 experimental days. The different stressors are represented with a dot if they are punctual or with a line if they last for a period. Abbreviations: R30, restriction during 30 min; BW, bedding wetting; R5, restriction during 30 min; LF, light flashes; CT, cage tilt; LFR, light flashes with restriction.

Animals with similar body weight were randomly assigned to two different groups (*n* = 10 animals per group): the CON group and the 3d CUMS group. The early stress approach is a short-adapted version of the classical CUMS model of behavioural stress to mimic a short stressor effect during the first 3 days of the CUMS model (Figure 8). Animals from the CON group were not subjected to any stress, and they were only handled to habituate them to the manipulator contact. Animals from the 3d CUMS group were subjected to different stressors for three consecutive days. The stressors included in the protocol were physical restriction for 30 min and 5 min; bedding wetting for 12 h (consisting of mixing 300 mL of water with 1 L of sawdust bedding); light flashes (120 flashes/minute for 15 s, followed by one minute of rest, and this procedure was repeated 5 times); 45° cage tilt for 15 h; and the combination of light flashes and physical restriction before the sacrifice.

### 4.2. OFT

To assess anxiety-like behaviour, at the end of the 3d CUMS (7 h before sacrifice) rats were individually placed in a grey wooden box (70 × 45 × 45 cm) and allowed to explore it for 5 min. A central area (20 × 40 cm) was considered for scoring time and number of entries in the inner zone. Locomotor activity, fecal boli deposits and rearing were also recorded and analysed using a tracking system (ANY-Maze, version 4.82, Stoelting Co., Wood Dale, IL, USA). The box was wiped clean with 70% ethanol before testing each animal.

### 4.3. Sample Collection

Urine samples were collected the day before the sacrifice through hydrophobic sand method, which is less stressful for the animals than others classical methodologies [53]. For each rat, 300 g of hydrophobic sand was spread (LabSand, Coastline Global, Palo Alto, CA, USA) on the bottom of a mouse plastic micro-isolation cage. Urine was collected every half hour for 6 h with sodium azide (Sigma, St. Louis, MO, USA) as preservative and was finally pooled at the end of the session. On the day of the sacrifice, animals were killed by guillotine under anaesthesia (sodium pentobarbital, 50 mg/kg per body weight) after 7 h of fasting. Blood was collected and centrifuged at 3000× *g* at 4 °C for 15 min to recover plasma. Tissues were rapidly removed, weighted and snap-frozen in liquid nitrogen (i.e., RWAT, MWAT, muscle, liver and cecum). All the samples were stored at −80 °C until further analysis.

### 4.4. Plasma Biochemistry

Concentrations of serotonin (#ab133053, Abcam, Cambridge, UK) and corticosterone (#EIACORT, Invitrogen, Carlsbad, CA, USA) were measured in plasma by ELISA according to the manufacturer’s protocol. Enzymatic colorimetric kits were used for the plasma determination of TC, TG, glucose (QCA, Barcelona, Spain) and NEFAs (WAKO, Neuss, Germany).

### 4.5. Metabolome Analysis

#### 4.5.1. Plasma Metabolome (GC-qTOF and UHPLC-qTOF)

Plasma metabolites were analysed by gas Chromatography coupled with Quadrupole Time-of-Flight (GC-qTOF). For the extraction, a protein precipitation extraction was performed by adding eight volumes of methanol:water (8:2, *v*/*v*) containing internal standard mixture (succinic acid-d4, myristic acid-d27, glicerol-13C3 and D-glucose-13C6) to plasma samples. Then, the samples were mixed and incubated at 4 °C for 10 min, centrifuged at 21,420× *g* and the supernatant was evaporated to dryness before compound derivatization (metoximation and silylation). The derivatized compounds were analysed by GC-qTOF (model 7200 of Agilent, Santa Clara, CA, USA). The chromatographic separation was based on the Fiehn Method, using a J&W Scientific HP5-MS (30 m × 0.25 mm i.d.), 0.25 µm film capillary column and helium as carrier gas using an oven program from 60 °C to 325 °C. Ionization was performed by electronic impact (EI), with electron energy of 70 eV and operated in full Scan mode. The identification of metabolites was performed matching two different parameters to metabolomic Fiehn library (Agilent, Santa Clara, CA, USA): EI mass spectrum, considered stable and reproducible and retention time. To avoid annotation errors, metabolites with very high molecular weights were cleared. After the putative identification of metabolites, these were semi-quantified in terms of the internal standard response ratio.

Plasma lipids were analysed by Ultra-High-Performance Liquid Chromatography coupled with Quadrupole Time-of-Flight (UHPLC-qTOF). For the extraction of the hydrophobic lipids, a liquid–liquid extraction based on the Folch procedure [54] was performed by adding four volumes of chloroform:methanol (2:1, *v*/*v*) containing internal standard mixture (Lipidomic SPLASH^®^, Avanti Polar Lipids, Inc., Alabaster, AL, USA) to plasma. Then, the samples were mixed and incubated at −20 °C for 30 min. Afterwards, water with NaCl (0.8%) was added, and the mixture was centrifuged at 21,420× *g*. Lower phase was recovered, evaporated to dryness and reconstituted with methanol:methyl-tert-butyl ether (9:1, *v*/*v*) and analysed by UHPLC-qTOF (model 6550 of Agilent, Santa Clara, CA, USA) in positive electrospray ionization mode. The chromatographic consists in an elution with a ternary mobile phase containing water, methanol, and 2-propanol with 10 mM ammonium formate and 0.1% formic acid. The stationary phase was a C18 column (Kinetex EVO C18 Column, 2.6 µm, 2.1 mm × 100 mm) that allows the sequential elution of the more hydrophobic lipids such as TGs, DGs PCs, ChoEs, LPCs and SMs, among others. The identification of lipid species was performed by matching their accurate mass and tandem mass spectrum, when available, to Metlin-PCDL from Agilent containing more than 40,000 metabolites and lipids. In addition, chromatographic behaviour of pure standards for each family and bibliographic information was used to ensure their putative identification. After putative identification of lipids, these were semi-quantified in terms of internal standard response ratio using one internal standard for each lipid family.

A pooled matrix of samples was generated by taking a small volume of each experimental sample serving as a technical replicate throughout the data set. As the study took multiple days, a data normalization step was performed to correct variation resulting from instrument inter-day tuning differences. Essentially, each compound was corrected in run-day blocks through quality controls, normalizing each data point proportionately.

#### 4.5.2. Urine Metabolome (^1^H-NMR)

Urine metabolites were analysed by proton nuclear magnetic resonance (^1^H-NMR). The urine sample was mixed (1:1, *v*/*v*) with phosphate buffered saline containing with 3-(Trimethylsilyl)propionic-2,2,3,3-d4 acid sodium salt (TSP) (Sigma Aldrich) and placed on a 5 nm NMR tube for direct analysis by ^1^H-NMR. ^1^H-NMR spectra were recorded at 300 K on an Avance III 600 spectrometer (Bruker^®^, Bremen, Germany) operating at a proton frequency of 600.20 MHz using a 5 mm PBBO gradient probe. Diluted urine aqueous samples were measured and recorded in procno 11 using one-dimensional ^1^H pulse experiments and were carried out using the nuclear Overhauser effect spectroscopy (NOESY). NOESY presaturation sequence (RD–90°–t1–90°–tm–90° ACQ) was used to suppress the residual water peak, and the mixing time was set at 100 ms. Solvent presaturation with irradiation power of 150 μW was applied during recycling delay (RD = 5 s) and mixing time (noesypr1d pulse program in Bruker^®^) to eliminate the residual water. The 90° pulse length was calibrated for each sample and varied from 11.21 to 11.38 ms. The spectral width was 9.6 kHz (16 ppm), and a total of 128 transients were collected into 64 k data points for each ^1^H spectrum. The exponential line broadening applied before Fourier transformation was of 0.3 Hz. The frequency domain spectra were manually phased and baseline-corrected using TopSpin software (version 3.2, Bruker). Data were normalized by two different ways, by the probabilistic method to avoid differences between samples due to different urine concentration and by ERETIC. The acquired ^1^H-NMR were compared to references of pure compounds from the metabolic profiling AMIX spectra database (Bruker^®^), HMDB and Chenomx databases for metabolite identification. In addition, we assigned metabolites by ^1^H-^1^H homonuclear correlation (COSY and TOCSY) and ^1^H-^13^C heteronuclear (HSQC) 2D NMR experiments and by correlation with pure compounds run in-house. After pre-processing, specific ^1^H-NMR regions identified in the spectra were integrated using MATLAB scripts run in-house. Curated identified regions across the spectra were exported to an Excel spreadsheet to evaluate robustness of the different ^1^H-NMR signals and to give relative concentrations.

### 4.6. Microbiome Analysis (Shotgun Metagenomic Sequencing)

The shotgun metagenomic sequencing was performed in 8 animals per group. DNA was extracted from cecum content using the PowerSoil DNA extraction kit (MO BIO Laboratories, Carlsbad, CA, USA) following the manufacturer’s protocol. Between 400 and 500 ng of total DNA was used for library preparation for Illumina sequencing employing Illumina DNA Prep kit (Illumina, San Diego, CA, USA). All libraries were assessed using a TapeStation High Sensitivity DNA kit (Agilent Technologies, Santa Clara, CA, USA) and quantified by Qubit (Invitrogen, Waltham, MA, USA).

Validated libraries were pooled in equimolar quantities and sequenced as a paired-end 150-cycle run on an Illumina NextSeq2000. A total of 1548 million reads were generated, and raw reads were filtered for QV > 30 using an in-house phyton script. Filtered reads were aligned to unique clade-specific marker genes using MetaPhlAn 3 [55] to assess the taxonomic profile. The alignment was performed indicating the closest name of species to the sequence (the best hit). The relative proportions calculated from MetaPhlAn were used to calculate relative abundances, alpha diversity measure (chao1 index) and beta diversity measure (Aitchison distance).

### 4.7. Statistical Analysis

#### 4.7.1. General Statistical Analysis

The statistical analysis was performed using the R statistical software (version 4.0.2, R Core Team 2021) and different libraries included in Bioconductor (version 3.11, Bioconductor project) were used [56]. The continuous variables of biological assay were shown as mean ± standard error of the mean (SEM) per group. After the normality study, parametric unpaired t-test was used for single statistical comparisons; thus, a two-tailed value of *p*-value < 0.05 was considered.

#### 4.7.2. Metabolomic Data Analysis

For metabolomics, the MW test was performed in this case because the variables follow the assumption of a non-parametric test. The *p*-value adjustment for multiple comparisons was carried out according to the BH correction considering a 5% of FDR. The magnitude of difference between populations was presented as fold change (FC) relative to the control group. In parallel, a predictive analysis was conducted to evaluate the prediction power of the experimental model. On the one hand, PCA, an unsupervised multivariate data projection method, was performed to explore the native relationship between groups. On the other hand, OPLS-DA, a supervised multivariate data projection method, was calculated to explore the possible relationships between the observable variables (X) and the predicted variables or target (Y), reflecting the variation in the data set. No data transformation has been applied before conducting the analysis. The predictive performance of the test set was estimated by the Q2Y parameter calculated through cross-validation. The values of Q2 < 0 suggests a model with no predictive ability, 0 < Q2 < 0.5 suggests some predictive character and Q2 > 0.5 indicates good predictive ability [57]. The feature importance was calculated through the VIP values that reflect both the loading weights for each component and the variability in the response explained by the component.

In this case, we followed a pipeline, which was previously described [58,59], considering statistical significance and predicting capability of individual metabolites to capture the major differences in the experimental study: (1) the metabolites reflecting general metabolic differences were selected according to the MW statistical test (*p*-value < 0.05) and the VIP (>1.0) values to present an overview of the impact in the metabolism of early stress stage; (2) the individual discriminating metabolites associated with stress and controls were selected primarily according to the MW statistical test (*p*-value < 0.01) and the BH correction (*q*-value < 0.05) and the VIP (>1.5) values to propose candidate biomarkers.

Additionally, RF was calculated to sort the most important metabolites that distinguish between the CON and 3d CUMS groups. The whole data set was used without rejecting the metabolites where no differences were observed using the MW test. Therefore, the 10 most relevant metabolites were presented.

#### 4.7.3. Metagenomic Data Analysis

For microbiome data, centred log-ratio (CLR) was performed before any statistical test. The beta diversity was calculated from the Aitchison distance, and PERMANOVA test was performed with 100 permutations to assess the differences between groups (*n* = 8 animals per group). The alpha diversity was calculated by Chao1 index. Taxonomic abundances were compared between experimental groups using the BH adjustment on MW test that is presented by relative abundance (%). The relative abundance was filtered to only include variables that were present above 0.01% in at least 3 samples [60]. The magnitude of difference between populations was determined by the determination of FC.

#### 4.7.4. Integration Data Analysis

For multi-omics data integration, DIABLO implementation in the mixOmics R package (version 6.18.1, mixOmics project) was used to integrate plasma and urine metabolome and microbiome [61]. The R script was added as supplementary data with the steps to perform all the integration analysis. To summarize, the first step is the parameter choice of the design matrix, the number of components and the number of variables to select: (1) a design matrix of 0.1 was used to focus primarily on the discrimination between the groups; (2) the perf function was used to estimate the performance of the model and the balanced error rate (BER) and overall error rates per component were displayed to select the optimal number of components; and (3) the number of variables was chosen using the tune.block.splsda function that is run with 10-fold cross validation and repeated 10 times, thus this tuning step led to a selection of 8 plasma metabolites, 6 urine metabolites and 5 microbes. Thereafter, the final model was computed, and different sample and variable plots were performed. In Figure 6a, the correlations greater than 0.7 between variables of different types were represented using the function circosPlot. In Figure 6b, the resulting network, which was calculated with the network function, was further analysed using Cytoscape (version 3.8.2, Institute of Systems Biology, Seattle, WA, USA) [62].

The final performance of the model was evaluated by the perf function using 10-fold cross-validation repeated 10 times. Additionally, the ROC curve analysis was conducted to evaluate the metabolite combination patterns that could correctly dichotomize the stressed and healthy groups at acceptable sensitivity and specificity (defined as greater than 80% for both). The AUC value was used as a measure of the prognostic accuracy, thus, an AUC value of 1 indicates a perfect test due to the absence of overlap of the test data between the groups. In this case, an AUC value above >0.85 was considered for inclusion in the model.

#### 4.7.5. Pathway Analysis

Finally, a comparison between univariate (MW test adjusted by BH) and multivariate analyses (PCA and OPLS-DA), RF and multi-omics integration (DIABLO) was performed to check the robustness among methods of the candidate biomarkers. The weights of the methods can be 0 (no influence) or 1 (influence), and the final value is a summatory of the weights of the different methods of analysis: Features with a weight of 3 (positive in all the methods) presented the highest impact on the model followed by weights of 2. The key features were analysed through different databases to identify related pathways and elucidate the global effect in the metabolism of an early stress stage. The main database consulted was the Kyoto Encyclopaedia of Genes and Genomes (KEGG) [62], among others. To show those results, XMind (version XMind 2020, XMind Ltd., Virginia, ON, Canada) was used to incorporate the information about pathway analysis (Figure 7).

## 5. Conclusions

In summary, differences between the groups were observed in the behaviour, biochemical parameters and metabolic patterns. Specifically, an omics profile was elucidated and was composed by a signature of eight plasma metabolites (threonic acid, alpha-ketoglutarate, malic acid, 3-hydroxybutiric acid, DG 34:2, succinic acid, aspartic acid and cholesterol); six urine metabolites (taurine, α-hydroxyhippurate, N-acetylglycine, malic acid, N,N-dimethylglycine and betaine); and five microbes, including bacteria and viruses (*Escherichia coli*, *Lactobacillus murinus*, *Akkermansia muciniphila*, uncharacterized *herpesvirus* and *Bacteroides uniformis*). Finally, seven metabolites may be considered a metabolic footprint from plasma (malic acid, threonic acid, alpha-ketoglutarate, succinic acid, cholesterol and pyruvic acid) and urine metabolomics (N,N-dimethylglycine). Furthermore, the full matching metabolites are malic acid, threonic acid, alpha-ketoglutarate, succinic acid and cholesterol in plasma metabolomics, these being the key metabolites of the early stress stage based on a 3d CUMS approach targeting the TCA cycle and energy metabolism. In addition, more studies profiling the early stress stage are recommended for the further exploration and validation of these omics profiles, metabolic footprints and potential candidate biomarkers.

## Figures and Tables

**Table 1:** Biometric and plasma parameters of the early stress stage. Data are presented as the mean ± SEM (*n* = 10 animals per group). (*) represent statistically significant differences among groups (*p* < 0.05) using Student’s *t*-test (*p*-value), and the FC represents the change magnitude. Abbreviations: RWAT, retroperitoneal white adipose tissue; MWAT, mesenteric white adipose tissue; TG, triglycerides; TC, total cholesterol; NEFAs, non-esterified fatty acids.

		CON	3d CUMS	*p*-Value	FC
Biometric parameters	Initial body weight (g)	476.67 ± 10.99	467.29 ± 12.04	0.57	0.98
	Final body weight (g)	476.50 ± 10.69	468.26 ± 10.44	0.59	0.98
	Food intake (g)	21.23 ± 0.76	20.71 ± 0.7	0.63	0.98
	RWAT weight (g)	11.33 ± 1.22	12.11 ± 1.3	0.67	1.07
	MWAT weight (g)	5.96 ± 0.52	7.28 ± 0.73	0.16	1.22
	Muscle weight (g)	2.97 ± 0.33	2.84 ± 0.45	0.67	0.96
	Liver weight (g)	12.26 ± 0.43	11.51 ± 0.3	0.17	0.94
	Cecum weight (g)	4.95 ± 0.26	4.51 ± 0.22	0.22	0.91
Plasma biochemistry	Corticosterone (ng/mL)	58 ± 6.6	374.5 ± 24.8	<0.01 *	6.46
	Serotonin (ng/mL)	49.99 ± 9.95	211.55 ± 50.95	0.01 *	4.32
	Glucose (mM)	67.56 ± 1.71	82.63 ± 2.60	<0.01 *	1.22
	TG (mM)	71.15 ± 4.34	82.75 ± 7.89	0.2	1.16
	TC (mM)	67.12 ± 2.92	79.30 ± 5.09	0.06	1.18
	NEFAs (mM)	0.42 ± 0.03	0.56 ± 0.04	0.02 *	1.33

**Table 2:** Summary of the significant differential plasma metabolites in the early stress stage. CON and 3d CUMS groups (*n* = 10 animals per group) are represented by the relative abundances (AU). Relative abundances of metabolites are presented by the mean ± SEM. Plasma metabolites are sorted by *p*-value. The summary of the analysis is shown and includes the relative abundances of metabolites, *p*-value, *q*-value, VIP value, RF, FC, the effect of the 3d CUMS versus the CON group and the related metabolic pathway.

Metabolite	CON	3d CUMS	*p*-Value	*q*-Value	VIP	RF	FC	Effect	Metabolic Pathway
Malic acid	0.36 ± 0.03	0.74 ± 0.07	<0.01	0.03	2.4	0.03	2.1	↑	TCA cycle
Threonic acid	2.55 ± 0.21	0.8 ± 0.17	<0.01	0.03	2.6	0.03	0.3	↓	Ascorbate and aldarate metabolism
Alpha-ketoglutarate	1.21 ± 0.08	1.94 ± 0.13	<0.01	0.03	2.3	0.03	1.6	↑	TCA cycle
Succinic acid	0.61 ± 0.04	0.86 ± 0.04	<0.01	0.03	2.3	0.03	1.4	↑	TCA cycle
Pyruvic acid	14.68 ± 0.85	25.39 ± 3.14	<0.01	0.03	2.1	0.03	1.7	↑	Glycolysis
Cholesterol	0.33 ± 0.02	0.6 ± 0.05	<0.01	0.05	2.4	0.05	1.8	↑	Steroid biosynthesis

## Data Availability

The data presented in this study are available on request from the corresponding author. The data are not publicly available due to interest of performing more analysis for further publications together with more data.

## Supplementary Materials

The following are available online at https://www.mdpi.com/article/10.3390/ijms222312931/s1.
