# Supplementary material for: Alterations in Metabolome and Microbiome Associated with an Early Stress Stage in Male Wistar Rats: A Multi-Omics Approach
Source: Int J Mol Sci. 2021 Nov 29;22(23):12931. doi: 10.3390/ijms222312931 (PMC8657954; doi:10.3390/ijms222312931)
Supplement: Supplementary file 1 [file ijms-22-12931-s001.zip › ijms-1463210-supplementary.pdf]

Supplementary Table S1. Statistical analysis of plasma metabolites in the early stress stage. CON and 3d CUMS groups (n = 10 animals per group) are represented by relative abundances (AU). Relative abundances of metabolites are presented by the mean ± SEM. Plasma metabolites are sorted by p-value. The summary of univariant and multivariate analysis is shown including p-value, q-value, VIP value, RF and FC. The statistically significant p-values and q-values are highlighted in bold. Abbreviations: DG, diacylglycerol; ChoE, cholesterol ester; TG, triglyceride; PC, phosphatidylcholine; SM, sphingomyelin; LPC, lysophospholipid; PE, phosphatidylethanolamine.

Metabolite	CON	3d CUMS	p-value	q-value	VIP	RF	FC
Malic acid	0.36 ± 0.03	0.74 ± 0.07	**<0.01**	**0.03**	2.42	0.23	2.1
Threonic acid	2.55 ± 0.21	0.8 ± 0.17	**<0.01**	**0.03**	2.63	0.15	0.3
Alpha-ketoglutarate	1.21 ± 0.08	1.94 ± 0.13	**<0.01**	**0.03**	2.30	0.10	1.6
Succinic acid	0.61 ± 0.04	0.86 ± 0.04	**<0.01**	**0.03**	2.25	0.04	1.4
Pyruvic acid	14.68 ± 0.85	25.39 ± 3.14	**<0.01**	**0.03**	2.09	0.05	1.7
Cholesterol	0.33 ± 0.02	0.6 ± 0.05	**<0.01**	**0.05**	2.38	0.05	1.8
Oleic acid	1.71 ± 0.09	2.47 ± 0.22	**<0.01**	0.06	1.80	0.12	1.4
3-hydroxybutiric acid	2.03 ± 0.19	2.76 ± 0.08	**0.01**	0.09	1.91	0.04	1.4
Citric acid	3.04 ± 0.14	3.8 ± 0.2	**0.01**	0.10	1.89	0.01	1.3
Phenylalanine	0.87 ± 0.15	0.55 ± 0.11	**0.01**	0.16	1.44	0.01	0.6
Threonine	1.35 ± 0.13	0.97 ± 0.11	**0.01**	0.16	1.56	-	0.7
DG 34:2	1 ± 0.05	0.84 ± 0.05	**0.02**	0.19	1.28	0.01	0.8
Glucose	0.78 ± 0.04	0.9 ± 0.04	**0.02**	0.19	1.53	0.01	1.2
Valine	1.69 ± 0.64	0.94 ± 0.42	**0.02**	0.19	0.85	-	0.6
Aspartic acid	0.53 ± 0.1	0.68 ± 0.04	**0.03**	0.21	1.49	0.02	1.3
Fructose	0.4 ± 0.02	0.3 ± 0.02	**0.03**	0.21	1.94	-	0.8
Glyceric acid	1.75 ± 0.12	1.33 ± 0.11	**0.03**	0.24	1.84	0.02	0.8
2-hydroxyglutaric	0.69 ± 0.07	0.51 ± 0.02	**0.05**	0.32	1.56	-	0.7
Asparagine	0.17 ± 0.02	0.24 ± 0.02	**0.05**	0.32	1.55	-	1.4
ChoE (18:2)	13.81 ± 0.53	15.18 ± 0.6	**0.05**	0.32	1.13	-	1.1
ChoE (18:3)	1.27 ± 0.09	1.74 ± 0.18	**0.05**	0.32	1.19	0.02	1.4
Hydroxyproline	0.54 ± 0.1	0.7 ± 0.07	**0.05**	0.32	1.35	0.01	1.3
Methionine	0.12 ± 0.02	0.17 ± 0.02	0.08	0.44	1.41	-	1.4
Tyrosine	0.9 ± 0.15	0.72 ± 0.09	0.09	0.49	0.57	-	0.8
Beta-alanine	0.08 ± 0.01	0.1 ± 0.02	0.10	0.52	0.80	-	1.3
TG 46:0	0.77 ± 0.05	0.66 ± 0.03	0.10	0.52	1.35	-	0.9
ChoE (16:0)	1.8 ± 0.06	1.97 ± 0.09	0.12	0.52	0.93	-	1.1
Histidine	0.14 ± 0.01	0.11 ± 0.01	0.12	0.52	1.10	-	0.8
Ornithine	3.12 ± 0.6	3.71 ± 0.36	0.12	0.52	0.90	-	1.2
Proline	0.26 ± 0.02	0.29 ± 0.01	0.12	0.52	1.06	0.02	1.1
Serine	0.26 ± 0.02	0.22 ± 0.01	0.12	0.52	1.06	-	0.8
ChoE (22:6)	1.67 ± 0.12	2.08 ± 0.17	0.14	0.54	1.38	-	1.2
DG 36:4	1.97 ± 0.13	1.88 ± 0.07	0.14	0.54	0.09	-	1
TG 52:1	0.98 ± 0.46	0.45 ± 0.05	0.14	0.54	1.32	-	0.5
TG 54:2	1.06 ± 0.52	0.48 ± 0.05	0.14	0.54	1.22	-	0.5
Glucose-6-phosphate	0.14 ± 0.02	0.18 ± 0.02	0.16	0.55	0.94	-	1.3
Glutamic acid	0.09 ± 0.01	0.12 ± 0.01	0.16	0.55	1.36	-	1.3
Lysine	1 ± 0.17	0.76 ± 0.1	0.16	0.55	0.71	-	0.8
PC 42:4 e	0.01 ± 0	0.01 ± 0	0.16	0.55	0.66	-	1
SM 33:1	0.34 ± 0.02	0.38 ± 0.02	0.16	0.55	0.92	-	1.1
ChoE (18:0)	0.11 ± 0.02	0.14 ± 0.01	0.19	0.60	0.89	-	1.3

TG 50:0	0.44 ± 0.13	0.28 ± 0.02	0.19	0.60	1.25	-	0.6
LPC 16:1 e	0.13 ± 0	0.14 ± 0	0.21	0.65	0.99	-	1.1
LPC 15:0	0.68 ± 0.03	0.74 ± 0.04	0.24	0.65	0.81	-	1.1
LPC 16:0	67.34 ± 1.75	71.03 ± 1.93	0.24	0.65	0.81	-	1.1
PC 33:1	0.04 ± 0.01	0.06 ± 0.01	0.24	0.65	0.83	-	1.5
PC 34:1	3.07 ± 0.26	3.59 ± 0.28	0.24	0.65	0.63	-	1.2
SM 34:2	1.65 ± 0.07	1.75 ± 0.1	0.24	0.65	0.44	-	1.1
SM 43:1	1.13 ± 0.04	1.03 ± 0.05	0.24	0.65	1.15	0.01	0.9
TG 52:5	13.23 ± 6.44	6.29 ± 1.23	0.24	0.65	1.18	-	0.5
LPC 18:2	31.95 ± 1.27	34.19 ± 1.03	0.27	0.67	0.80	-	1.1
TG 46:1	0.67 ± 0.15	0.5 ± 0.03	0.27	0.67	1.12	-	0.7
TG 48:1	1.64 ± 0.43	1.22 ± 0.17	0.27	0.67	1.03	-	0.7
TG 54:3	6.28 ± 3.07	2.83 ± 0.34	0.27	0.67	1.19	-	0.5
Fumaric acid	1.04 ± 0.15	1.25 ± 0.1	0.31	0.67	0.91	-	1.2
PC 35:2	0.38 ± 0.02	0.4 ± 0.02	0.31	0.67	0.26	-	1.1
Ribose	4.05 ± 0.33	3.66 ± 0.39	0.31	0.67	0.88	-	0.9
SM 35:1	0.16 ± 0.01	0.17 ± 0.01	0.31	0.67	0.80	-	1.1
TG 48:0	1.24 ± 0.31	0.86 ± 0.04	0.31	0.67	1.19	-	0.7
TG 54:4	17.8 ± 8.18	8.52 ± 1.28	0.31	0.67	1.17	-	0.5
Tryptophan	1.75 ± 0.26	1.55 ± 0.26	0.31	0.67	0.44	-	0.9
ChoE (20:4)	62.3 ± 3.02	67.79 ± 3.27	0.34	0.67	0.92	-	1.1
ChoE (22:4)	4.72 ± 0.26	5.18 ± 0.3	0.34	0.67	0.59	-	1.1
PE 38:5 e	2.07 ± 0.36	2.64 ± 0.56	0.34	0.67	0.63	-	1.3
SM 32:1	0.24 ± 0.01	0.27 ± 0.02	0.34	0.67	0.47	-	1.1
TG 46:2	0.39 ± 0.08	0.3 ± 0.02	0.34	0.67	1.05	-	0.8
TG 51:2	1.03 ± 0.45	0.58 ± 0.08	0.34	0.67	1.01	-	0.6
TG 52:3	52.41 ± 16.39	33.45 ± 5.31	0.34	0.67	1.09	-	0.6
TG 52:6	1.91 ± 1.01	0.94 ± 0.22	0.34	0.67	1.04	-	0.5
TG 54:7	8.71 ± 4.11	4.34 ± 0.75	0.34	0.67	1.16	-	0.5
Alanine	0.39 ± 0.07	0.45 ± 0.08	0.38	0.70	0.63	-	1.2
ChoE (18:1)	2.35 ± 0.07	2.43 ± 0.18	0.38	0.70	0.09	-	1
PC 34:3 e	0.02 ± 0	0.02 ± 0	0.38	0.70	0.83	-	1
ChoE (17:0)	0.13 ± 0.01	0.13 ± 0	0.43	0.70	0.48	-	1
ChoE (20:2)	0.86 ± 0.05	0.91 ± 0.06	0.43	0.70	0.08	-	1.1
DG 36:2	1.39 ± 0.06	1.45 ± 0.06	0.43	0.70	0.66	-	1
LPC 18:0	49.81 ± 1.79	51.54 ± 2.09	0.43	0.70	0.35	-	1
PC 36:2	12.64 ± 0.81	13.17 ± 0.67	0.43	0.70	0.17	-	1
TG 50:2	15.37 ± 7.15	8.76 ± 1.9	0.43	0.70	0.95	-	0.6
TG 52:2	21.51 ± 13.25	8.94 ± 1.76	0.43	0.70	0.84	-	0.4
TG 54:6	16.71 ± 3.96	12.25 ± 1.56	0.43	0.70	1.04	-	0.7
ChoE (17:1)	0.07 ± 0	0.07 ± 0	0.47	0.70	0.40	-	1
Fructose-6-phosphate	0.14 ± 0.02	0.16 ± 0.02	0.47	0.70	0.38	-	1.1
PC 32:0	0.58 ± 0.03	0.6 ± 0.03	0.47	0.70	0.11	-	1
PC 38:6 e	0.05 ± 0	0.06 ± 0	0.47	0.70	0.81	-	1.2
SM 34:1	17.31 ± 0.65	18.33 ± 0.97	0.47	0.70	0.57	-	1.1
SM 36:1	1.14 ± 0.05	1.21 ± 0.07	0.47	0.70	0.54	-	1.1
SM 36:2	0.42 ± 0.02	0.44 ± 0.02	0.47	0.70	0.63	-	1
TG 48:2	2.31 ± 1.29	1.17 ± 0.25	0.47	0.70	0.87	-	0.5
TG 48:3	0.7 ± 0.37	0.37 ± 0.07	0.47	0.70	0.87	-	0.5
TG 50:1	6.36 ± 3.98	2.51 ± 0.48	0.47	0.70	0.96	-	0.4

TG 50:4	2.91 ± 1.73	1.3 ± 0.32	0.47	0.70	0.94	-	0.4
Alpha-tocopherol	0.56 ± 0.04	0.53 ± 0.03	0.52	0.73	0.45	-	0.9
ChoE (22:5)	0.55 ± 0.03	0.58 ± 0.04	0.52	0.73	0.25	-	1.1
LPC 20:0	0.31 ± 0.01	0.32 ± 0.01	0.52	0.73	0.53	-	1
PC 34:1 e	0.11 ± 0	0.11 ± 0.01	0.52	0.73	0.41	-	1
PC 36:4	15.99 ± 0.86	16.57 ± 0.89	0.52	0.73	0.12	-	1
PC 38:3	0.61 ± 0.12	0.68 ± 0.08	0.52	0.73	0.47	-	1.1
DG 34:3	0.37 ± 0.02	0.4 ± 0.03	0.57	0.76	0.50	-	1.1
LPC 18:1	12.68 ± 0.59	13.55 ± 0.61	0.57	0.76	0.66	-	1.1
PC 32:1	0.26 ± 0.04	0.3 ± 0.06	0.57	0.76	0.03	-	1.2
PC 36:2 e	0.01 ± 0	0.01 ± 0	0.57	0.76	0.68	-	1
DG 34:1	1.14 ± 0.03	1.12 ± 0.03	0.62	0.77	0.38	-	1
PC 40:4	0.13 ± 0.01	0.14 ± 0.01	0.62	0.77	0.07	-	1.1
SM 38:1	0.49 ± 0.02	0.51 ± 0.03	0.62	0.77	0.32	-	1
SM 39:1	0.16 ± 0.03	0.14 ± 0.02	0.62	0.77	0.75	-	0.9
SM 40:1	3.58 ± 0.08	3.83 ± 0.18	0.62	0.77	0.70	-	1.1
SM 40:2	0.79 ± 0.06	0.81 ± 0.06	0.62	0.77	0.07	-	1
SM 42:1	13.2 ± 0.37	13.47 ± 0.68	0.62	0.77	0.07	-	1
TG 50:3	11.1 ± 6.87	5.07 ± 1.39	0.62	0.77	0.85	-	0.5
ChoE (16:1)	0.38 ± 0.03	0.4 ± 0.03	0.68	0.81	0.18	-	1.1
Isoleucine	0.71 ± 0.32	0.41 ± 0.19	0.68	0.81	0.34	-	0.6
LPC 18:0 e	0.08 ± 0	0.08 ± 0	0.68	0.81	0.12	-	1
PE 36:4	3.24 ± 0.31	2.98 ± 0.3	0.68	0.81	0.68	-	0.9
Lactic acid	6.9 ± 0.46	7.11 ± 0.36	0.73	0.85	0.40	-	1
PC 32:2	0.15 ± 0.02	0.16 ± 0.01	0.73	0.85	0.10	-	1.1
PC 38:2	0.11 ± 0.01	0.09 ± 0.01	0.73	0.85	0.92	-	0.8
PC 38:4 e	0.05 ± 0	0.05 ± 0	0.73	0.85	0.10	0.01	1
Leucine	0.26 ± 0.12	0.14 ± 0.08	0.79	0.89	0.51	-	0.5
PC 33:0	0.03 ± 0	0.03 ± 0	0.79	0.89	0.48	-	1
SM 42:2	8.38 ± 0.2	8.27 ± 0.47	0.79	0.89	0.34	-	1
Urea	2.94 ± 0.11	2.86 ± 0.12	0.79	0.89	0.42	-	1
Glycine	2.74 ± 0.55	2.25 ± 0.36	0.85	0.92	0.37	-	0.8
PC 36:3 e	0.05 ± 0	0.05 ± 0	0.85	0.92	0.13	-	1
PC 38:5 e	0.09 ± 0.01	0.09 ± 0.01	0.85	0.92	0.05	-	1
PC 40:5	0.16 ± 0.03	0.14 ± 0.01	0.85	0.92	0.45	-	0.9
SM 41:1	3.49 ± 0.09	3.59 ± 0.14	0.85	0.92	0.28	-	1
Glutamine	1.13 ± 0.17	1.03 ± 0.19	0.91	0.95	0.29	-	0.9
Glycolic acid	3.79 ± 0.12	4 ± 0.35	0.91	0.95	0.02	-	1.1
PC 30:0	0.04 ± 0	0.04 ± 0	0.91	0.95	0.41	-	1
PC 36:0	0.09 ± 0	0.09 ± 0.01	0.91	0.95	0.15	-	1
LPC 16:0 e	0.39 ± 0.02	0.39 ± 0.02	0.97	0.98	0.10	-	1
PC 38:4	19.45 ± 1.2	19.4 ± 0.93	0.97	0.98	0.14	-	1
SM 41:2	0.66 ± 0.04	0.65 ± 0.04	0.97	0.98	0.23	-	1
SM 42:3	3.95 ± 0.16	3.97 ± 0.17	0.97	0.98	0.07	-	1
PC 31:0	0.03 ± 0	0.03 ± 0	1.00	1.00	0.82	-	1
PC 34:0	0.31 ± 0.01	0.32 ± 0.02	1.00	1.00	0.05	-	1

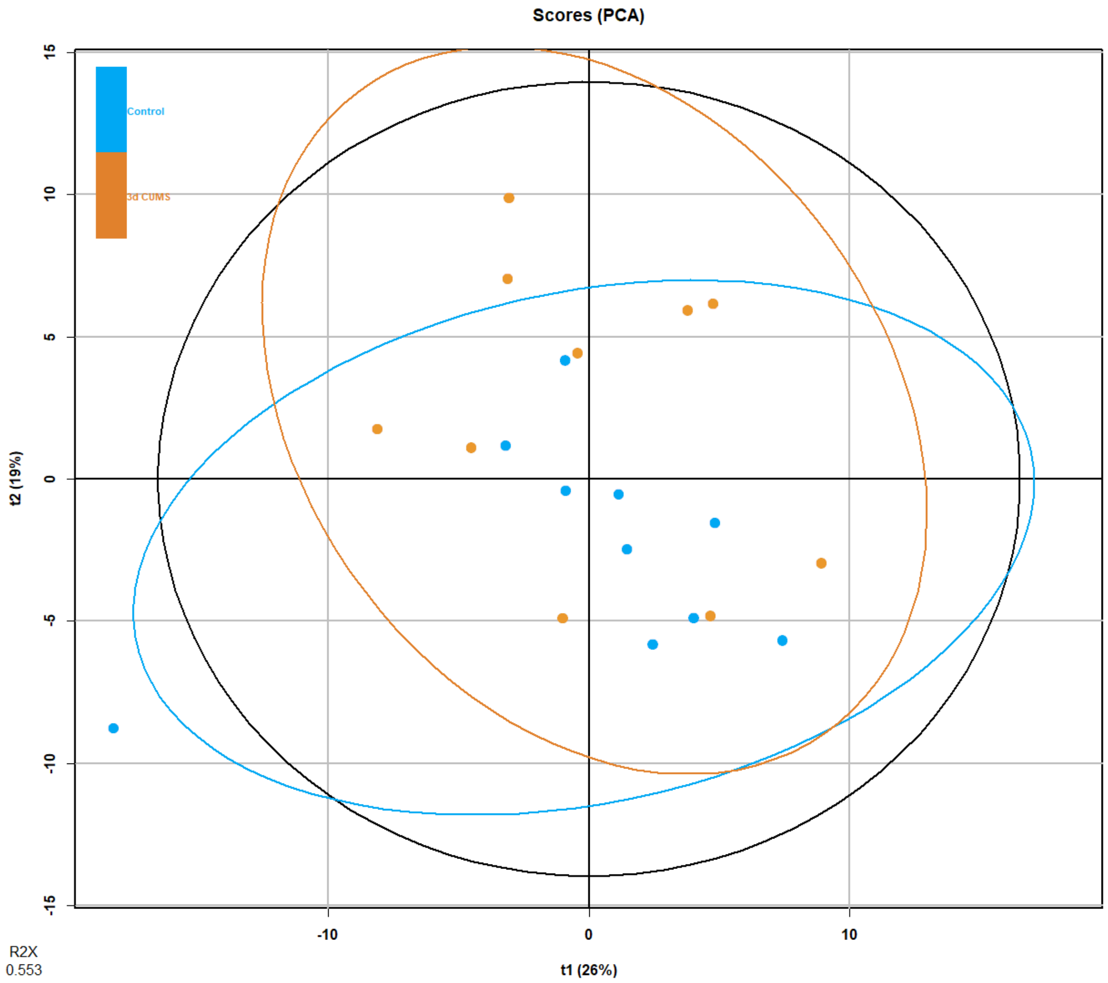

Supplementary Figure S1. PCA of plasma metabolomics in the early stress stage. Blue represents CON group and orange 3d CUMS group (n = 10 animals per group).

Supplementary Table S2. Statistical analysis of urine metabolites in the early stress stage. CON and 3d CUMS groups (n = 10 animals per group) are represented by relative abundances (AU). Relative abundances of metabolites are presented by the mean ± SEM. Urine metabolites are sorted by p-value. The summary of univariant and multivariate analysis is shown including p-value, q-value, VIP value and FC. The statistically significant p-values and q-values (< 0.05) are highlighted in bold. Abbreviations: TMAO, trimethylamine N-oxide; 3-HPPA, 3-hydroxyphenylpropionate; 4-PY, methyl-4-pyridone-5-carboxamide; DMA, Dimethylamine; NAD+, nicotinamide adenine dinucleotide.

Metabolite	CON	3d CUMS	p-value	q-value	VIP	RF	FC
Taurine	442.78 ± 38.45	644.38 ± 53.46	**0.01**	0.38	2.57	0.18	1.5
N.N-Dimethylglycine	3.71 ± 0.59	2.18 ± 0.21	**0.02**	0.38	2.27	0.21	0.6
Methylamine	5.09 ± 0.44	4.15 ± 0.37	0.08	0.88	1.52	0.07	0.8
N-Acetylglycine	29.45 ± 1.25	31.9 ± 2.26	0.11	0.88	0.69	0.07	1.1
Glycine	7.75 ± 0.65	6.39 ± 0.43	0.13	0.88	1.78	0.04	0.8
TMAO	3 ± 0.21	2.52 ± 0.16	0.13	0.88	1.98	-	0.8
Pseudouridine	9.09 ± 0.81	9.42 ± 0.68	0.21	0.88	0.08	-	1
2-Oxoglutarate	292.51 ± 36.51	244.45 ± 36.43	0.24	0.88	0.72	0.09	0.8
Betaine	28.54 ± 3.05	32.05 ± 2.64	0.24	0.88	0.50	-	1.1
Malate	6.94 ± 1.01	8.84 ± 0.73	0.24	0.88	1.21	-	1.3

Alanine	4.3 ± 0.48	3.56 ± 0.27	0.31	0.88	1.31	-	0.8
Hippurate	235.99 ± 18.29	211.23 ± 13.23	0.31	0.88	1.12	0.02	0.9
α-hydroxyhippurate	0.74 ± 0.1	0.89 ± 0.11	0.31	0.88	0.59	-	1.2
Citrate	336.66 ± 36.14	320.26 ± 32.95	0.35	0.88	0.12	0.01	1
Phenylacetylglycine	34.25 ± 6.33	40.12 ± 4.67	0.35	0.88	0.39	0.01	1.2
N-acetylglycoprteins	63.31 ± 2.81	64.9 ± 4.13	0.39	0.88	0.02	0.05	1
Trimethylamine	0.51 ± 0.14	0.89 ± 0.25	0.39	0.88	1.62	0.05	1.7
Tyrosine	7.43 ± 1.49	8.06 ± 1.24	0.39	0.88	0.09	0.03	1.1
NAD+	0.35 ± 0.07	0.31 ± 0.05	0.44	0.90	0.47	0.01	0.9
2-deoxycytidine	1.24 ± 0.16	1.36 ± 0.16	0.49	0.90	0.49	-	1.1
Creatinine	185.21 ± 10.84	166.45 ± 11.89	0.49	0.90	1.54	-	0.9
Leucine	13.3 ± 0.73	12.25 ± 0.83	0.49	0.90	1.15	0.01	0.9
Formate	2.33 ± 0.37	2.08 ± 0.28	0.54	0.90	0.44	-	0.9
Fumarate	3.02 ± 0.45	2.57 ± 0.43	0.54	0.90	0.60	0.01	0.9
Succinate	41.36 ± 2.33	43.83 ± 3.83	0.54	0.90	0.68	-	1.1
1-methylnicotinamide	0.11 ± 0.08	0.07 ± 0.03	0.60	0.90	0.43	0.04	0.6
Allantoin	196.28 ± 11.94	181.57 ± 10.82	0.60	0.90	1.25	0.02	0.9
o-Coumaric acid	1.57 ± 0.4	1.77 ± 0.39	0.65	0.90	0.30	-	1.1
Sarcosine	10.39 ± 1.27	9.73 ± 1.09	0.65	0.90	0.60	-	0.9
Dimethylamine (DMA)	14.68 ± 3.03	11.5 ± 1.22	0.71	0.90	0.16	0.01	0.8
Indoxyl Sulphate	5.19 ± 0.4	5.5 ± 0.6	0.71	0.90	0.13	-	1.1
3-methyl-2-oxovalerate	4.92 ± 0.39	5.7 ± 0.83	0.78	0.90	0.69	-	1.2
N6-Acetyllysine	30.2 ± 2.44	31.7 ± 2.03	0.78	0.90	0.37	-	1
3-HPPA	4.86 ± 2.09	2.77 ± 0.71	0.84	0.90	0.96	-	0.6
3-hydroxyisovalerate	3.65 ± 0.33	3.53 ± 0.25	0.84	0.90	0.40	-	1
4-PY	5.86 ± 0.64	6.31 ± 0.39	0.84	0.90	0.38	-	1.1
Acetate	5.31 ± 0.76	6.29 ± 1.19	0.84	0.90	0.57	-	1.2
HPPA sulfate	3.44 ± 0.95	2.63 ± 0.37	0.84	0.90	0.94	-	0.8
Tryptophan	5.21 ± 0.4	5.5 ± 0.6	0.84	0.90	0.08	-	1.1
Valine	1.16 ± 0.1	1.32 ± 0.2	0.84	0.90	0.45	-	1.1
Lactate	11.55 ± 1.09	11.25 ± 0.7	0.90	0.94	0.26	-	1
Fucose	8.57 ± 0.55	8.48 ± 0.5	1.00	1.00	0.39	0.01	1

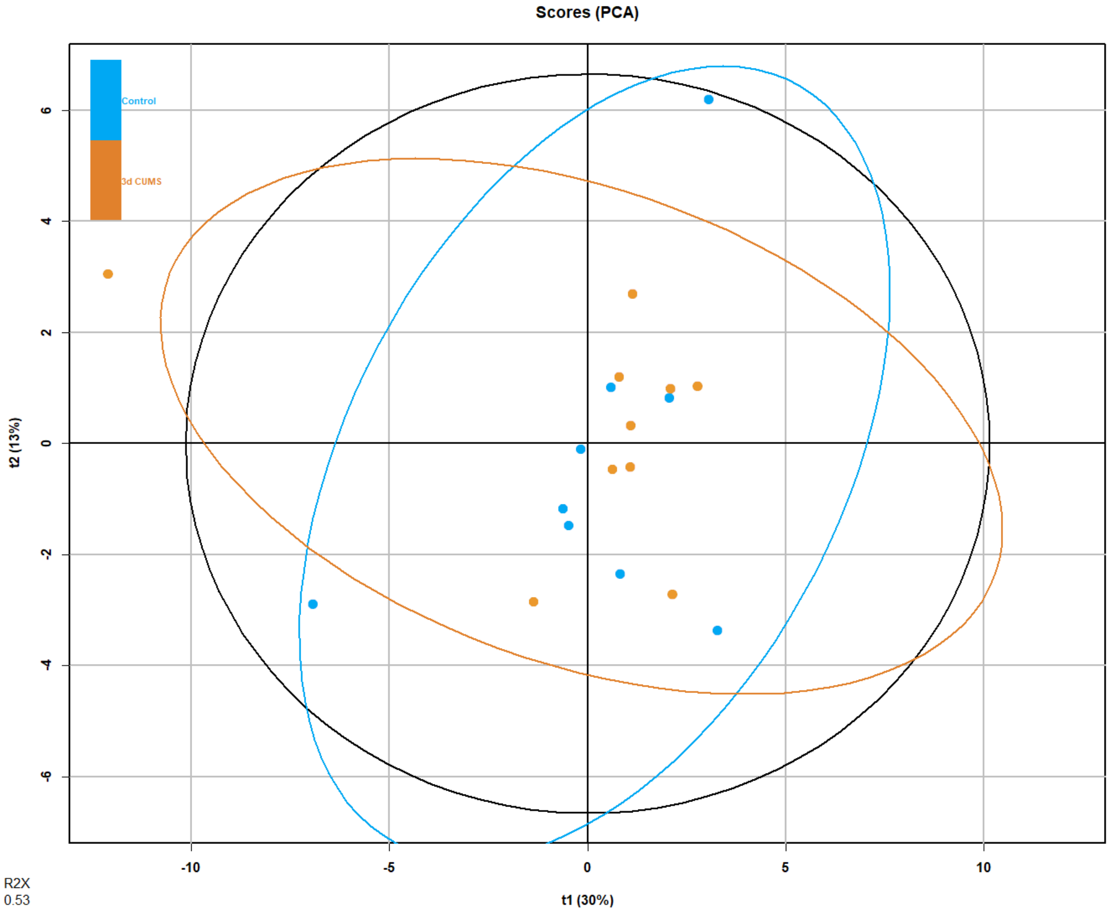

Supplementary Figure S2. PCA of urine metabolomics in the early stress stage. Blue represents CON group and orange 3d CUMS group (n = 10 animals per group).

Supplementary Table S3. Summary of bacteria species in the early stress stage. The summary of univariant analysis includes the results of MW test (p-value), MW corrected by BH (q-value) and FC; between CON group and 3d CUMS group (n = 8 animals per group). The alignment was done indicating the closest name of specie to the sequence (the best hit). Taxonomic data is presented by the mean of relative abundance (%) and shorted by p-value. The statistically significant values (< 0.05) are highlighted in bold.

Specie	CON	3d CUMS	p-value	q-value	FC
Bacteroides uniformis	7.71%	4.93%	**0.05**	0.32	0.6
Escherichia coli	6.27%	1.75%	**0.05**	0.32	0.3
Lactobacillus murinus	10.68%	2.24%	0.06	0.32	0.2
Akkermansia muciniphila	25.84%	41.66%	0.11	0.42	1.6
Firmicutes bacterium ASF500	0%	0.03%	0.28	0.43	10.0
Muribaculaceae bacterium DSM 103720	24.09%	25.33%	0.30	0.43	1.1
Muribaculum intestinale	14.31%	13.61%	0.30	0.43	1.0
Mucispirillum schaedleri	5.26%	3.81%	0.34	0.43	0.7
Bacteroides caecimuris	0.02%	0.03%	0.34	0.43	1.7
Anaerotruncus sp G3 2012	0.89%	0.98%	0.41	0.44	1.1
Oscillibacter sp 1 3	0.46%	0.41%	0.41	0.44	0.9
Parabacteroides goldsteinii	4.47%	5.21%	0.48	0.48	1.2

Supplementary Table S4. Summary of virus species in the early stress stage. The summary of univariant analysis includes results of MW test (p-value), MW corrected by BH (q-value) and FC; between CON group and 3d CUMS group (n = 8 animals per group). The alignment was done indicating the closest name of specie to the sequence (the best hit). Taxonomic data is presented by the mean of relative abundance (%) and shorted by p-value. The statistically significant values (< 0.05) are highlighted in bold.

**Specie**	**CON**	**3d CUMS**	**p-value**	**q-value**	**FC**
uncharacterized *herpesvirus*	38.50%	61.01%	**0.02**	0.31	1.6
Human alphaherpesvirus 2	0%	0.33%	**0.05**	0.40	-
Alcelaphine gammaherpesvirus 1	5.94%	3.05%	0.07	0.40	0.5
Lactobacillus prophage Lj928	0.14%	2.02%	0.13	0.40	14.9
Koala retrovirus	0.33%	0.06%	0.25	0.50	0.2
Lactobacillus prophage Lj771	7.11%	5.48%	0.30	0.50	0.8
Abelson murine leukemia virus	13.90%	6.14%	0.34	0.50	0.4
Pestivirus Giraffe 1	0.18%	0.30%	0.34	0.50	1.6
Murine osteosarcoma virus	9.29%	6.15%	0.41	0.50	0.7
Ateline gammaherpesvirus 3	5.29%	3.62%	0.43	0.50	0.7
Stx2 converting phage 1717	6.93%	4.40%	0.45	0.50	0.6
Bovine alphaherpesvirus 1	0.33%	0.78%	0.47	0.50	2.4
Anguillid herpesvirus 1	3.57%	4.21%	0.50	0.50	1.2

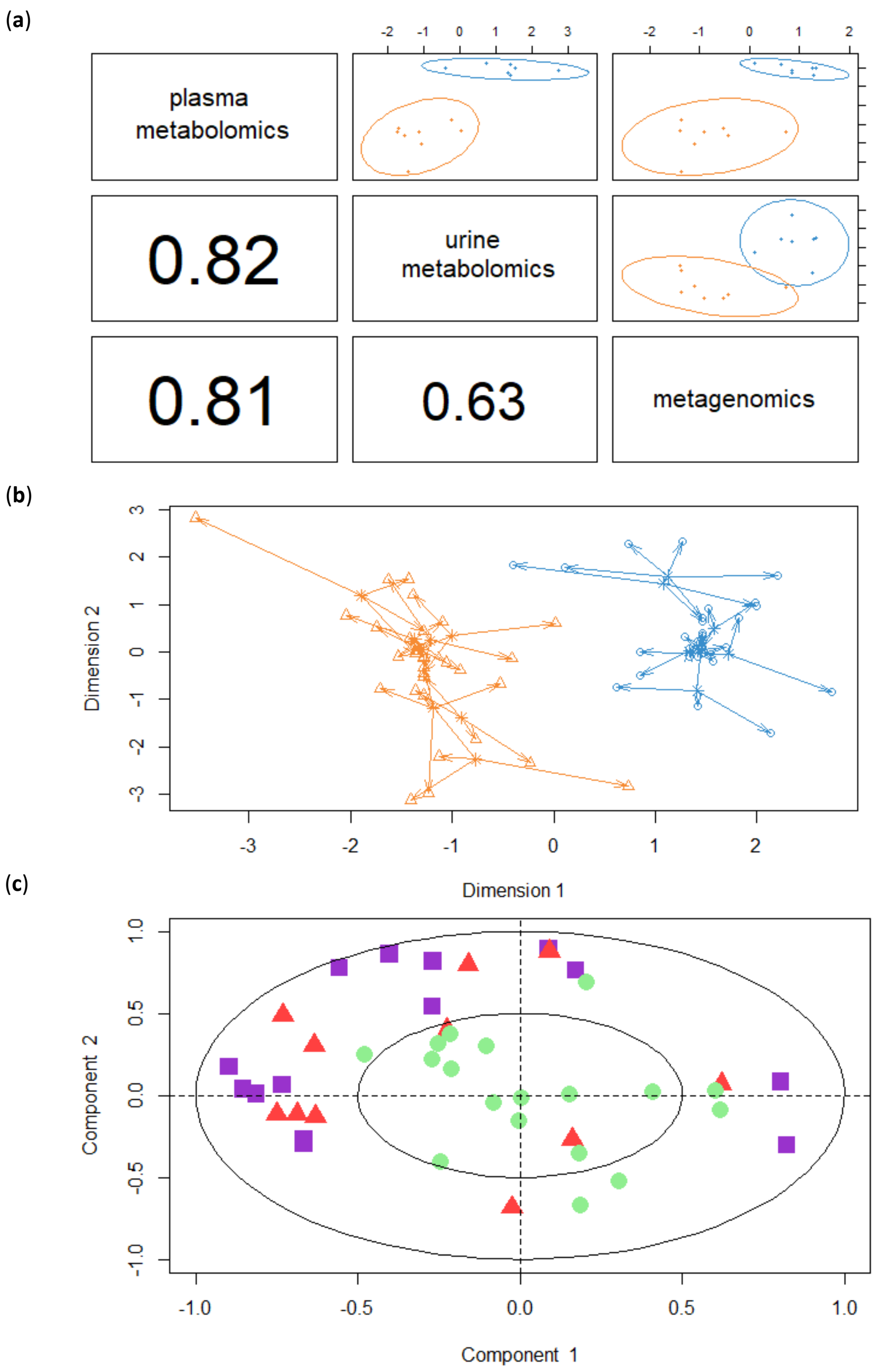

Supplementary Figure S3. Integration of plasma metabolomics, urine metabolomics and metagenomics data using DIABLO in the early stress stage. (a) Global overview: correlation values between data at the component level (component 1). The first components from each data set are highly correlated to each other. (b) Arrow plot: the start of the arrow indicates the centroid between all data sets for a given sample and the tips of the arrows the location of that sample in each omic, highlighting the agreement between the 3 data sets at the sample level. In the figure (a) and (b) the group 3d CUMS is represented by orange and the CON group by blue. (c) Correlation circle plot: This plot highlights the contribution of each selected variable to component 1 and 2. Clusters of points indicate a strong correlation between variables. Each colour and shape indicate the type of features, i.e., plasma metabolites (purple square), urine metabolites (red triangle) and finally, bacteria and virus species (green circle). 3d CUMS in orange and CON in blue.

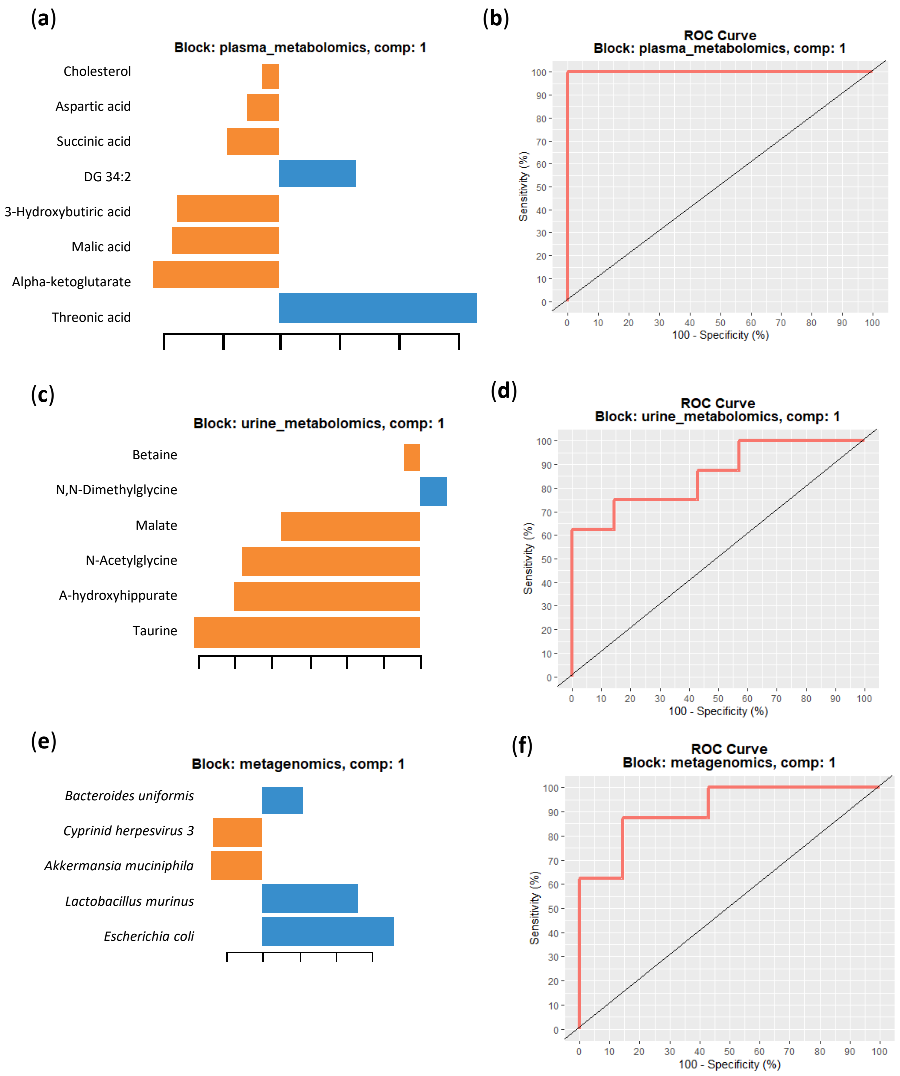

Supplementary Figure S4. Feature integration in plasma metabolomics, urine metabolomics and metagenomics in the early stress stage. (a) (c) (e) Feature impact in each data set in component 1. 3d CUMS in orange and CON in blue. (b) (d) (f) ROC curve and AUC averaged using one-vs-all comparisons in the different data set.

Supplementary Table S5. Summary of the comparison between univariate and multivariate analysis, random forest, and multi-omics integration in the early stress stage. The different methods of analysis are represented in the table - univariate and multivariate (U/M) analysis, random forest (RF) and the multi-omics integration (DIABLO) – and the weights of the features in the different omic approaches. The values of the methods can be 0 (no influence) or 1 (influence). The final value is the summatory of the weights of the different methods of analysis: features with weight of 3 (green) presented the highest impact on the model followed by weights of 2 (yellow). Abbreviations: DG, diacylglycerol; ChoE, cholesterol ester.

Source	Feature	U/M analysis	RF	DIABLO	Weight
Plasma metabolomics	Malic acid	1	1	1	3
Plasma metabolomics	Threonic acid	1	1	1	3
Plasma metabolomics	Alpha-ketoglutarate	1	1	1	3
Plasma metabolomics	Succinic acid	1	1	1	3
Plasma metabolomics	Cholesterol	1	1	1	3
Plasma metabolomics	3-hydroxybutiric acid	0	1	1	2

Plasma metabolomics	Pyruvic acid	1	1	0	2
Plasma metabolomics	Oleic acid	0	1	0	1
Plasma metabolomics	ChoE (18:3)	0	1	0	1
Plasma metabolomics	Glyceric acid	0	1	0	1
Plasma metabolomics	Aspartic acid	0	0	1	1
Plasma metabolomics	DG 34:2	0	0	1	1
Urine metabolomics	N,N-Dimethylglycine	0	1	1	2
Urine metabolomics	Taurine	0	1	1	2
Urine metabolomics	2-Oxoglutarate	0	1	0	1
Urine metabolomics	N-Acetylglycine	0	0	1	1
Urine metabolomics	Methylamine	0	1	0	1
Urine metabolomics	Trimethylamine	0	1	0	1
Urine metabolomics	N-acetylglycoprteins	0	1	0	1
Urine metabolomics	2-oxoglutarate	0	1	0	1
Urine metabolomics	1-methylnicotinamide	0	1	0	1
Urine metabolomics	Glycine	0	1	0	1
Urine metabolomics	α-hydroxyhippurate	0	0	1	1
Urine metabolomics	Malic acid	0	0	1	1
Urine metabolomics	Betaine	0	0	1	1
Metagenomics	*Escherichia coli*	0	0	1	1
Metagenomics	*Lactobacillus murinus*	0	0	1	1
Metagenomics	*Akkermansia muciniphila*	0	0	1	1
Metagenomics	uncharacterized *herpesvirus*	0	0	1	1
Metagenomics	*Bacteroides uniformis*	0	0	1	1
